# Aquaporin-Based Biomimetic Polymeric Membranes: Approaches and Challenges

**DOI:** 10.3390/membranes5030307

**Published:** 2015-07-31

**Authors:** Joachim Habel, Michael Hansen, Søren Kynde, Nanna Larsen, Søren Roi Midtgaard, Grethe Vestergaard Jensen, Julie Bomholt, Anayo Ogbonna, Kristoffer Almdal, Alexander Schulz, Claus Hélix-Nielsen

**Affiliations:** 1 Technical University of Denmark, Department of Environmental Engineering, Miljøvej, Building 113, 2800 Kgs. Lyngby, Denmark; E-Mail: joachim.habel@gmx.de; 2 Aquaporin A/S, Ole Maaløes Vej 3, 2200 Copenhagen, Denmark; E-Mails: jbo@aquaporin.dk (J.B.); annyogbonna@yahoo.com (A.O.); 3 University of Copenhagen, Department of Plant and Environmental Sciences, Thorvaldsensvej 40, 1871 Frederiksberg, Denmark; E-Mails: mh@plen.ku.dk (M.H.); als@plen.ku.dk (A.S.); 4 University of Copenhagen, Copenhagen Biocenter, Ole Maaløes Vej 5, 2200 Copenhagen, Denmark; E-Mails: kynde@nbi.ku.dk (S.K.); soromi@nbi.ku.dk (S.R.M.); gvjensen@nbi.ku.dk (G.V.J.); 5 University of Copenhagen, Niels Bohr Institute, Hans Christian Ørsted building D, Universitetsparken, 5, 2100 Copenhagen, Denmark; E-Mail: nanna.skaarup@gmail.com; 6 Technical University of Denmark, Department of Micro- and Nanotechnology, Produktionstorvet, Building 423, 2800 Kgs. Lyngby; E-Mail: kral@nanotech.dtu.dk; 7 University of Maribor, Laboratory for Water Biophysics and Membrane Processes, Faculty of Chemistry and Chemical Engineering, Smetanova ulica 17, 2000 Maribor, Slovenia

**Keywords:** biomimetic membranes, aquaporins, block copolymers, proteopolymersomes, polyhedral oligomeric silsesquioxanes, polyamide layer, microfluidics, membrane proteins, protein-polymer-interactions

## Abstract

In recent years, aquaporin biomimetic membranes (ABMs) for water separation have gained considerable interest. Although the first ABMs are commercially available, there are still many challenges associated with further ABM development. Here, we discuss the interplay of the main components of ABMs: aquaporin proteins (AQPs), block copolymers for AQP reconstitution, and polymer-based supporting structures. First, we briefly cover challenges and review recent developments in understanding the interplay between AQP and block copolymers. Second, we review some experimental characterization methods for investigating AQP incorporation including freeze-fracture transmission electron microscopy, fluorescence correlation spectroscopy, stopped-flow light scattering, and small-angle X-ray scattering. Third, we focus on recent efforts in embedding reconstituted AQPs in membrane designs that are based on conventional thin film interfacial polymerization techniques. Finally, we describe some new developments in interfacial polymerization using polyhedral oligomeric silsesquioxane cages for increasing the physical and chemical durability of thin film composite membranes.

## 1. Introduction

Aquaporin biomimetic membranes (ABMs) have attracted interest recently due to their potentially superior performance in terms of water flux and solute rejection as compared to conventional membranes. Their superior performance has been demonstrated experimentally [1,2,3]. The number of Web of Science entries for biomimetic membranes has increased by a factor of four from 2000 to 2011. The general ABM approach has been reviewed recently [4,5,6]. Basically, the ABMs developed until now are made from combining three components: Aquaporins (AQPs) which are membrane protein water channels; amphiphilic molecules in which the AQPs are embedded; and a polymer support structure. The amphiphilic molecules generally can be either lipids or polymers. Due to superior performance in terms of stability and flexibility [7], block copolymers (di- or triblock) have been predominantly investigated resulting in aquaporin-based biomimetic polymeric membranes (ABPMs), but a number of studies also address aquaporin-based biomimetic lipidic membranes (ABLMs).

Here, we attempt to provide a broad overview of how AQPs, block copolymers and polymer support structures interact and how these interactions can be characterized. In the first section, we will discuss the interplay between AQPs and block copolymers including general membrane protein incorporation into block copolymers, resulting in AQP-block copolymer complexes in vesicular (proteopolymersomes) or planar form. Many aspects of AQP incorporation were lessons learnt from the study of incorporation of other membrane proteins. We then review characterization methods for studying proteopolymersomes including freeze-fracture transmission electron microscopy (FF-TEM), stopped-flow light scattering (SFLS), fluorescence correlation spectroscopy (FCS) and small-angle X-ray scattering (SAXS). In order to fabricate membranes, the reconstituted AQPs need to be integrated in a suitable supporting matrix. Here, we will describe recent advances with emphasis on how to understand the interplay between proteopolymersomes and polymer-based supporting structures in the form of polyamide active layer (PA-AL) formation. PA-based thin film composite membranes represent a classical approach to membrane fabrication. However, it remains a challenge to control stability, surface roughness and other material properties of the PA-AL. We have therefore investigated if proteo- and polymersomes can be integrated in PA-AL containing polyhedral oligomeric silsesquioxanes (POSS) and how the AL is influenced by this integration in terms of physical and chemical stability and surface roughness. POSS is a well-defined nano-scale organic-inorganic structure that allows for constructing nano-structured hybrid materials and nanocomposites. With respect to membrane technology, POSS has been investigated in terms of creating membranes for molecular separation at elevated temperatures [8] and membranes with anti-fouling properties [9]. However, it is not clear if POSS is compatible with proteopolymersome incorporation. Here, we briefly describe methods to investigate POSS-proteopolymersome interactions using a microfluidic approach for membrane formation [10].

## 2. Interactions between Aquaporin Proteins and Block Copolymer Matrixes

Although most work on membrane protein incorporation has been performed with lipids as host matrix components (first proteoliposomes publication appeared in 1971 [11]), polymer-based incorporation has gained considerable interest since the first proteopolymersomes publication appeared in 2000 [12]. The early work focused on incorporation of membrane-spanning proteins including ATPases and bacteriorhodopsin into polymethyloxazoline-polydimethylsiloxane-polymethyloxazoline (PMOXA-PDMS-PMOXA) triblock copolymer bilayers in planar [13] or vesicular form [14,15,16].

It is intriguing that membrane proteins can be incorporated functionally in polymeric bilayers (e.g., based on PMOXA-PDMS-PMOXA) that can be up to 10 times thicker than their lipidic counterparts [17]. In fact, proteopolymersomes have been observed with protein densities that exceed proteoliposomes by far [18].

A theoretical approach has been established for general membrane protein incorporation into amphiphilic structures. In this approach, the membrane protein incorporation efficiency depends on its hydrophobicity and its coupling to the host membrane, which is directly related to hydrophobic mismatch. To minimize the mismatch, the host membrane has to deform to match the hydrophobic length of the transmembrane segment of the membrane protein about 3–4 nm. The alternative mode of adaption, a host membrane-induced membrane protein deformation is unlikely as far as the compressibility of membrane proteins is generally one to two orders of magnitude higher than lipids [19]. For polymers, the compression-expansion modulus is assumed to rise linearly with increasing molecular weight (*M_w_*), in which chain compression is favorable over chain stretching. This linear increase is consistent with the notion that the hydrophobic mismatch energy can be balanced with a decrease in stretching energy in the polymer chains around the incorporated membrane protein [20]. Srinivas and Discher found by using coarse-grain simulations that flexible hydrophobic chains can allow protein incorporation, even when the hydrophobic mismatch between membrane protein and hydrophobic interior of the chain region is greater than 22% [21,22]. Thus, membrane proteins can be incorporated more effectively if the hydrophobic chains are flexible [20]. Because flexible chains may however block the channel, no functionality of proteopolymersomes might be observed, even if the membrane protein has been incorporated functionally [21]. Moreover, high polydispersity can as well lead to a higher incorporation efficiency because the shorter chains can gather around the membrane protein and compensate for the hydrophobic mismatch. The good incorporation observed with PMOXA-PDMS-PMOXA could therefore also be attributed to their significantly high polydispersity index (*P DI*). In contrast, for natural lipid environment, the annual lipids around the incorporated protein can be selected in part by affinity to the protein surface and lateral diffusion [23]. The effect of hydrophobic mismatch is significant for ATPases, co-transporter proteins and ion channels [19], whereas for AQPs, the effects appear smaller—likely because the protein itself is structurally more rigid [24].

The first incorporation of AQPs in polymer bilayer was done in 2004 by Stoenescu and coworkers [25]. They incorporated AQP0 that is derived from the mammalian eye-lens, in polymersomes of three different block architectures (ABA, ABC, CBA, where A stands for PMOXA, B for PDMS and C for polyethylene oxide, PEO). The block configuration dictates the orientation of the incorporated AQP0. Where ABA had 50% of incorporated AQP0 with an orientation similar to that observed in liposomes, CBA had only 20% and ABC 80%, as evidenced by antibody labeling. In all cases, incorporation is achieved by adding AQP0 in detergent during the polymersome formation and removing the non-incorporated protein by size exclusion chromatography (SEC) [25].

The first demonstration of functional AQP incorporation was presented by Kumar in 2007 who incorporated bacterial AqpZ from *E.coli* in PMOXA-PDMS-PMOXA polymersomes [17] and proved their functionality within SFLS. SFLS is a common permeability characterization method, in which the polymersome shrinkage due to a response to osmolarity change is monitored over time by light scattering. Incorporation of AqpZ led to 800 times higher osmotic response of proteopolymersomes compared to empty polymersomes and showed that the activation energy, meaning the barrier for water to pass through the AqpZ, was comparable to that of AQP reconstituted in proteoliposomes and frog oocytes. The molar protein-to-amphiphile-ratio (mPAR) for optimal AqpZ performance in the triblock copolymer system was found to be 1:50 which would correspond to 1:100 in a (diblock- or lipid) bilayer system [17]. The high density reconstitution of AQP is further exemplified by the formation of 2D AQP crystals to achieve structural (crystallographic) information about AQP—analogous to what has been done with lipid based 2D AQP crystals [26]. For this purpose, a monolayer of nickel-functionalized polybutadiene-polyethylene oxide (PB-PEO) is accumulated at the water-interface, where in the aqueous solution, mixed micelles of detergent, histidine-tagged AqpZ and PDMS-PMOXA-PDMS were present [27]. The nickel affinity to the histidine binds the AqpZ to the PB-PEO layer [28], facilitating a high packing of AqpZ in this layer. After removing the detergent via biobeads and the PB-PEO via imidazole, densely packed AqpZ PMOXA-PDMS-PMOXA crystals were left, unfortunately not of sufficient quality to obtain any structural information [29,30].

**Figure 1:**
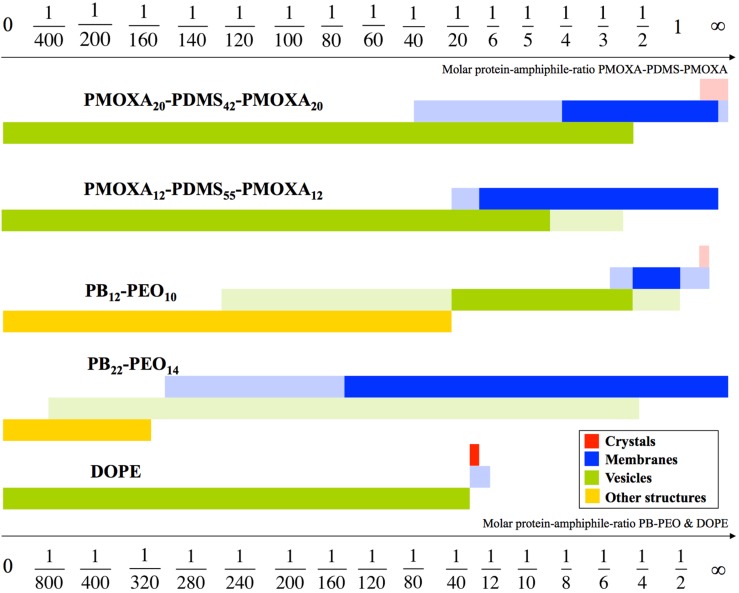
Schematic drawing of aggregate morphologies as a function of mPAR. PB_12_-PEO_10_ undergoes four transitions. Surprisingly, the vesicle shape remained at significantly higher densities at block copolymers, compared to a standard lipid like 1,2-dioleoyl-sn-glycero-3-phosphoethanolamine (DOPE). The mPAR of the one-molecule-bilayer-forming ABA triblock copolymers was divided by two enabling direct comparison with the PB-PEO diblock copolymers and DOPE, both forming bilayers. The morphologies in full color are the main morphologies, pale colors denote coexisting morphologies. Adapted from [34].

**Figure 2:**
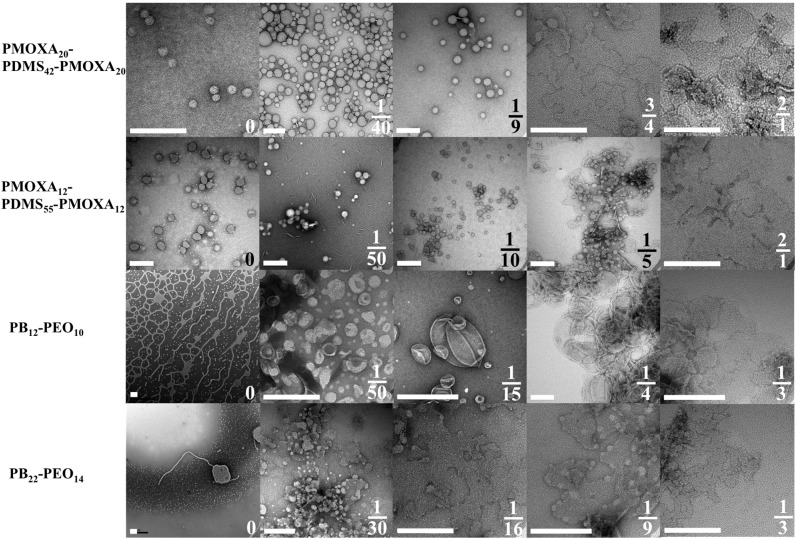
TEM images of aggregate morphologies as a function mPAR. Where the PMOXA-PDMS-PMOXA copolymers self-assemble to vesicles, PB-PEO forms network- and sperm-like structures and only after incorporation of AQP0 vesicular structures are observed. Scale bar is 200nm. Adapted from [33].

2D crystals can in fact be used to investigate the influence of AQP on polymer self-assembly in general. AQP0 is known to easily form 2D crystals due to its natural occurrence in stacks in the eye lens [31]. The findings here were that AQP0 dictates the self-assembling behavior of both polymers in a reciprocal way to the hydrophilic volume ratio *f*. With increasing mPAR, the interfacial curvature decreases and polymersomes turn into membrane sheets and partly crystals (see Figure 1 and Figure 2). In the case of PB-PEO, formation of polymersomes only occurred by adding AQP0, whereas without AQP0 only cylindrical structures are observed. The highest measured packing densities of functional AQPs into vesicular structures are observed at PB-PEO polymersomes with an mPAR of 1:15, which is significantly higher than what has been achieved in proteoliposomes or frog oocytes. Although not all AQP0 protein was incorporated, the seven-fold increase in osmotic response is consistent with a high-packing density given the relatively low permeability of AQP0 [32]. In this case incorporation was done via mixing detergent-solubilized polymers with detergent-solubilized AQP0 and subsequently dialyzing out the detergent [18,33].

With respect to fabrication of biomimetic membranes for technological purposes the first protein incorporation approaches from 2009–2011 were mainly lipid based [35,36], but also planar polymeric membranes have been demonstrated with functional incorporation of gramicidin A [37]. These efforts were pioneered by the Danish company Aquaporin A/S. Their later achievements in fabricating biomimetic membrane will be discussed in the next sections, as well as the work coming out of the groups at the Singapore Membrane Technology Center (SMTC) at Nanyang Technological University (NTU) and the National University of Singapore (NUS).

Table 1 summarizes all experimental membrane protein (and peptide) incorporations in block copolymer membranes, including polymer chemistry and stochiometry, *PDI*, the number-average molecular weight (*M_n_*), *f*, the incorporated membrane protein, the transport cargo (e.g., water for AQP), if there was functional incorporation, mPAR, the shape of the polymer self-assembled structure, how polymer and membrane protein were mixed and how the function incorporation was measured. *M_n_* (which can be quantified using NMR) is related to *M_w_* as *PDI* = *M_w_*/*M_n_*. The table excludes those incorporation studies which do not involve block copolymer-protein interactions, such as cell-free expression systems [38,39,40], encapsulation in hydrophobic interior [16], nanopores [41,42], non-amphiphilic polymers [43] and hydrogel approaches [44,45]. With this limitation, the table shows that most results were published by Wolfgang Meier and coworkers using on PMOXA-PDMS-PMOXA triblock copolymers.

**Table 1:** Overview of studies of membrane protein incorporation into amphiphilic block copolymers. Most studies are done with the porin OmpF, followed by AqpZ. For explanations please refer to the list of abbreviations.

Polymer	M*_n_*	PDI	*f*	Membrane Protein	Transport Cargo	FI?	mPAR	S	Incorporation method Main Functional incorporation measurement		References
PMOXA_13_–PDMS_23_–PMOXA_13_	3.9				e^−^	X	1:3300	V	MAq, biobeads & SEC	Cargo → Reduction of MP → EPR signal	[49]
PMOXA_13_–PDMS_23_–PMOXA_13_	4.7	NA	0.44	Alamethicin		X	1:590	P	MAq	Current change	[50]
PMOXA_13_–PDMS_23_–PMOXA_13_	4.7	NA	0.44	Hemolysin		X	1:110,000,000	P	MAq	Current change	[50]
PMOXA_13_–PDMS_23_–PMOXA_13_	4.7	NA	0.44	OmpG		X	1:33,000,000	P	MAq	Current change	[50]
PMOXA_20_-PDMS_41_-PMOXA_20_	6.4	1.61	0.49	NtAQP1	CO_2_	X	1:360	P	MOr	Cargo → Reaction inside vesicle → pH change	[51]
PMOXA_20_-PDMS_41_-PMOXA_20_	6.4	1.61	0.49	NtPIP2:1	CO_2_	X	1:360	P	MOr	Cargo → Reaction inside vesicle → pH change	[51]
PMOXA_20_-PDMS_41_-PMOXA_20_	6.5	<1.2	0.51	AQP0	H_2_O	ND	10:1–1:1	P	MAq & dialysis		[18]
PMOXA_20_-PDMS_41_-PMOXA_20_	6.5	<1.2	0.51	AQP0	H_2_O	ND	10:1–1:50	V	MAq & dialysis		[18]
PMOXA_20_-PDMS_41_-PMOXA_20_	6.5	<1.2	0.51	AQP0	H_2_O	-	1:2.5–0	V	MAq & dialysis	Vesicle size change	[18]
PMOXA_12_-PDMS_54_-PMOXA_12_	6.0	1.01	0.2	AqpZ	H_2_O	X	1:100–1:1600	V	MAq & biobeads	Vesicle size change	[3,52]
PMOXA_19_-PDMS_74_-PMOXA_19_	8.7	1.46	0.23								
PMOXA_12_-PDMS_54_-PMOXA_12_	6.0	1.01	0.30	AqpZ	H_2_O	X	1:50–1:400	V	MAq & biobeads	Vesicle size change	[53,54]
PMOXA_12_-PDMS_54_-PMOXA_12_	6.0	1.01	0.30	Hemolysin		-	1:83,000,000	P	MAq	Current change	[50]
PMOXA_20_-PDMS_54_-PMOXA_20_	7.4	NA	0.42	TsX	Nucleosides	X	1:450	V	MOr, SI & SEC	Cargo → Encapsulated enzyme activity → Color change	[55]
PMOXA_8_-PDMS_55_-PMOXA_8_	5.4	NA	0.22	AqpZ	H_2_O	X	1:3500	V	PFR, biobeads & SEC	Vesicle size change	[56]
PMOXA_12_-PDMS_55_-PMOXA_12_	6.1	1.64	0.30	OmpF	ELF97	X	1:1200	V	MAq & SEC Cargo	Precipitation inside vesicle → Color change	[57]
PMOXA_12_-PDMS_55_-PMOXA_12_	6.1	1.64	0.30	OmpF	Acridine orange	X	1:9,100,000	V	PPFR & SEC	Cargo release → Color change	[58]
PMOXA_12_-PDMS_55_-PMOXA_12_	6.1	1.64	0.30	OmpF	Paraquat. Pyocyanin	X	1:640	V	MAq & dialysis	No cargo → No detoxication of encapsulated enzyme*→* Cell death	[59,60]
PMOXA_12_-PDMS_55_-PMOXA_12_	6.1	1.64	0.30	AQP0	H_2_O	ND	10:1–1:25	P	MAq & dialysis		[18]
PMOXA_12_-PDMS_55_-PMOXA_12_	6.1	1.64	0.30	AQP0	H_2_O	-	1:3–0	V	MAq & dialysis	Vesicle size change	[18]
PMOXA_12_-PDMS_55_-PMOXA_12_	6.1	1.64	0.30	AqpZ	H_2_O	ND	1:4	Cr. V	MAq & biobeads		[29]
PMOXA_7_-PDMS_60_-PMOXA_7_	5.6	NA	0.19	Gramicidin A	Monovalent cations	X	1:81,000	P	MOr	Current change	[37]
PMOXA_8_-PDMS_60_-PMOXA_8_	5.8	NA	0.21	AqpZ	H_2_O	X	1:3800	V	PFR, biobeads & SEC	Vesicle size change	[56]
PMOXA_13_-PDMS_62_-PMOXA_13_	6.8	1.47	0.29	NADH reductase	e^−^	X	1:1900	V	MAq, biobeads & SEC	Cargo → Reduction of MP → EPR signal	[49]
PMOXA_15_-PDMS_62_-PMOXA_15_	7.1	1.50	0.32	NADH reductase	e^−^	X	1:1800	V	MAq, biobeads & SEC	Cargo → Reduction of MP → EPR signal	[49]
PMOXA_12_-PDMS_65_-PMOXA_12_	6.9	1.67	0.27	MloK1	Potassium	X	1:390	P	MAq & biobeads	Current change	[61]
PMOXA_15_-PDMS_68_-PMOXA_15_	7.6	NA	0.30	LamB	Maltohexaose	X	NA	P	MAq	Current change at varying cargo concentrations	[62]
PMOXA_15_-PDMS_68_-PMOXA_15_	7.6	NA	0.30	OmpF	Actylthiocholine	X	1:10000	V	PFR	Cargo → Encapsulated enzyme activity → Color change	[62]
PMOXA_15_-PDMS_68_-PMOXA_15_	7.6	1.20	0.30	AqpZ	H_2_O	X	1:10–1:1000	V	PFR & biobeads	Vesicle size change	[63]
PMOXA_15_-PDMS_68_-PMOXA_15_	7.6	1.20	0.30	Hemolysin			1:66,000,000	V	MAq	Current change	[50]
PMOXA_21_-PDMS_69_-PMOXA_21_	8.7	2.00	0.37	NADH reductase	e^−^	X	1:1500	V	MAq, biobeads & SEC	Cargo → Reduction of MP → EPR signal	[49]
PMOXA_16_-PDMS_72_-PMOXA_16_	8.0	1.17	0.30	OmpF	Enone	X	1:220	V	PPFR & dialysis	Cargo → Encapsulated enzyme activity → Color change	[64]
PMOXA-PDMS-PMOXA	8.8	NA	NA	OmpF	ELF97	X	1:50	V	MAq & SEC	Cargo → Precipitation inside vesicle → Color change	[65]
PMOXA_32_-PDMS_72_-PMOXA_32_	10.7	1.83	0.47	OmpF	7-ADCA. PGME	X	NA	V	PFR & dialysis	Cargo → Encapsulated enzyme activity → Bacterial death	[66]
PMOXA_11_-PDMS_73_-PMOXA_11_	7.2	1.70	0.22	LamB	DNA	X	1:390	V	MOr, SI & SEC	Fluorescence-labelled cargo	[67]
PMOXA_11_-PDMS_73_-PMOXA_11_	7.2	1.70	0.22	OmpF	Nucleosides	X	1:10, 1:100	V	PPFR & SEC	Cargo → Encapsulated enzyme activity → Color change	[68]
PMOXA_11_-PDMS_73_-PMOXA_11_	7.2	1.70	0.22	TsX	Nucleosides	X	1:10, 1:100	V	PPFR & SEC	Cargo → Encapsulated enzyme activity → Color change	[68]
PMOXA_11_-PDMS_73_-PMOXA_11_	7.2	1.70	0.22	LamB	DNA	X	NA	P	MAq		[67]
Lipids											
PMOXA_21_-PDMS_73_-PMOXA_21_	9.0	1.70	0.36	Alamethicin	Calcium	X	1:24	V	MAq	Cargo precipitation inside vesicle	[69,70]
PMOXA_21_-PDMS_73_-PMOXA_21_	9.0	1.70	0.36	FhuA	Sulphorhodamine B	X	1:6,000,000	V	MOr, SI & SEC	Cargo → Quenching inside vesicle → Color change	[71,72,73]
PMOXA_21_-PDMS_73_-PMOXA_21_	9.0	1.70	0.36	FhuA	TMB	X	1:4500. 1:3,600,000	V	MAq/ & biobeads/MOr, SI & SEC	Cargo → Encapsulated enzyme activity → Color change	[71,72,74]
PMOXA_21_-PDMS_73_-PMOXA_21_	9.0	1.70	0.36	FhuA		ND	3000:1	P	MAq		[72]
PMOXA_21_-PDMS_73_-PMOXA_21_	9.0	1.70	0.36	FhuA	NAD	-	NA	V	MAq	Cargo → Encapsulated enzyme activity*→* Absorbance change of cargo	[73]
PMOXA_21_-PDMS_73_-PMOXA_21_	9.0	1.70	0.36	FhuA	DNA	-	NA	V	MOr, SI & SEC	Fluorescence-labelled cargo	[73]
PMOXA_21_-PDMS_73_-PMOXA_21_	9.0	1.70	0.36	LamB	Sugar	X	NA	P	MAq	Current change at varying cargo concentration	[75]
PMOXA_21_-PDMS_73_-PMOXA_21_	9.0	1.70	0.36	OmpF	e^−^	X	NA	P	MAq	Current change	[75]
PMOXA_21_-PDMS_73_-PMOXA_21_	9.0	1.70	0.36	OmpF	Ampicillin	X	1:1000	V	MOr & SEC	Cargo → Hydrolysis inside vesicle → Color change	[12,76]
PMOXA_20_-PDMS_75_-PMOXA_20_	9.0	1.46	0.34	AqpZ	H_2_O	X	1:25, 1:50, 1:200	V	PFR & biobeads	Vesicle size change	[77]
PMOXA_11_-PDMS_76_-PMOXA_11_	7.8	1.48	0.25	BR	H^+^	X	NA	V/Mc	MOr & SI	pH change	[78,79]
PMOXA_11_-PDMS_76_-PMOXA_11_	7.8	1.48	0.25	BR & ATPase	H^+^	X	1:180	V	MOr & dialysis	pH change & bioluminescence assay	[15]
PMOXA_11_-PDMS_76_-PMOXA_11_	7.8	1.48	0.25	BR & ATPase	H^+^	X	1:20	V	PBR & dialysis	pH change	[80,81,82]
PMOXA_6_-PDMS_90_-PMOXA_6_	9.5	NA	0.12	OmpF	L-ascorbic acid, CO, Na_2_ S_2_ O_4_, ONOO^−^	X	1:1300	V	PFR, dialysis & SEC	Cargo → Absorbance change of encapsulated protein	[83]
PMOXA_21_-PDMS_97_-PMOXA_21_	9.0	1.70	0.30	Hemaglutinin		X	1:3800	V	MAq & biobeads	MP → Fusion with fluorescence-labelled liposomes	[74]
PMOXA_9_-PDMS_106_-PMOXA_9_	9.4	1.38	0.14	NADH reductase	e^−^	X	1:1400	V	MAq, biobeads & SEC	Cargo → Reduction of MP → EPR signal	[49]
PMOXA_13_-PDMS_110_-PMOXA_13_	10.4	1.44	0.19	NADH reductase	e^−^	X	1:1200	V	MAq, biobeads & SEC	Cargo → Reduction of MP → EPR signal	[49]
PMOXA_14_-PDMS_110_-PMOXA_14_	10.5	1.36	0.20	NADH reductase	e^−^	X	1:1200	V	MAq, biobeads & SEC	Cargo → Reduction of MP → EPR signal	[49]
PMOXA_15_-PDMS_110_-PMOXA_15_	10.7	1.62	0.21	AqpZ	H_2_O	X	1:25–1:500	V	PFR & SEC	Vesicle size change	[17,29]
PMOXA_15_-PDMS_110_-PMOXA_15_	10.7	1.62	0.21	OmpF		ND	NA	P	MAq		[84]
PMOXA-PDMS-PMOXA	20.0	NA		FhuA	Calcein	X	1:2,700,000	V	MOr, SI & SEC	Cargo release → Color change	[85]
PMOXA_65_-PDMS_165_-PMOXA_65_		23.3	1.63	NADH reductase	e^−^	X	1:550	V	MAq, biobeads & SEC	Cargo → Reduction of MP → EPR signal	[49]
PMOXA-PDMS-PMOXA	NA	NA	NA	BR	H^+^	X	NA	P	MAq	pH change	[86,87]
PMOXA-PDMS-PMOXA	NA	NA	NA	BR & CcO	H^+^ & e^−^	X	NA	V	MOr, SI & SEC	Current & pH change	[87,88]
PMOXA-PDMS-PMOXA	NA	NA	NA	CcO	e^−^	X	NA	P	MOr, SI & SEC	Current change	[86,87]
PMOXA-PDMS-PMOXA	NA	NA	NA	OmpF	H^+^	X	NA	P	MAq	Current change	[89]
PMOXA_110_-PDMS_40_-PEO_25_	13.4	NA	0.75	AQP0	H_2_O	ND	1:200	V	MOr, SI & SEC		[25]
PMOXA_45_-PDMS_40_-PMOXA_67_	10.6	NA	0.68	AQP0	H_2_O	ND	1:200	V	MOr, SI & SEC		[25]
MPEG-PVL	6.5	<1.2	0.00	Polymyxin B	Calcein	X	1:2	V	MAq	Cargo release → Color change	[73]
P2VP-PEO	NA	NA	NA	FhuA	NAD	-	NA	V	MOr, SI & SEC	Cargo → Enzyme reaction inside vesicle → Absorbance change of cargo	[73]
PB_12_-PEO_10_	1.1	1.09	0.32	AQP0	H_2_O	X	1:5–1:250	V	MAq & dialysis	Vesicle size change	[18]
PB_12_-PEO_10_	1.1	1.09	0.32	AQP0	H_2_O	ND	1:1.3	Cr	MAq & dialysis		[18]
PB_12_-PEO_10_	1.1	1.09	0.32	AQP0	H_2_O	ND	1:1–1:10	P	MAq & dialysis		[18]
PB_12_-PEO_10_	1.1	1.09	0.3	AqpZ	H_2_O	X	1:50-1:1000	V	MAq & dialysis	Vesicle size change	[90]
PB_12_-PEO_10_	1.1	1.09	0.32	BR	H^+^	X	1:500	V	MAq & biobeads	pH change	[91]
PB_12_-PEO_10_	1.1	1.09	0.3	SoPIP2;1	H_2_O	-	1:200	V	MAq & biobeads	Vesicle size change	[90]
PB_12_-PEO_10_	1.1	NA	0.34	Hemolysin	Calcein	X	1:33,000	V	MAq & dialysis	Cargo release → Color change	[92]
PB_22_-PEO_14_	1.8	1.17	0.28	AQP0	H_2_O	ND	2:1–1:300	P	MAq & dialysis		[18]
PB_22_-PEO_23_	2.2	1.09	0.39	AqpZ	H_2_O	X	1:15–1:200	V	MAq & dialysis	Vesicle size change	[90]
PB_22_-PEO_23_	2.2	1.09	0.39	SoPIP2;1	H_2_O	-	1:15, 1:200	V	MAq & dialysis	Vesicle size change	[90]
PB_29_-PEO_16_	2.3	1.00	0.25	AQP10	H_2_O	-	1:990	V	PFR & SE	Vesicle size change	-
PB_35_-PEO_14_	2.5	1.09	0.19	AqpZ	H_2_O	-	1:15	V	MAq & dialysis	Vesicle size change	[90]
PB_35_-PEO_14_	2.5	1.09	0.19	SoPIP2;1	H_2_O	-	1:15	V	MAq & dialysis	Vesicle size change	[90]
PB_43_-PEO_32_	3.7	1.03	0.31	AQP10	H_2_O	X	1:600	V	PFR & SE	Vesicle size change	[93]
PB_46_-PEO_30_	3.8	1.04	0.28	AqpZ	H_2_O	-	1:50,1:100,1:200	V	MAq & dialysis	Vesicle size change	[90]
PB_46_-PEO_32_	3.9	1.00	0.30	AQP10	H_2_O	-	1:580	V	PFR & SE	Vesicle size change	-
PB_52_-PEO_29_	4.1	<1.1	0.25	Hemolysin	e^−^	X	NA	P	MAq	Current change	[94]
PB_52_-PEO_29_	4.1	<1.1	0.25	Polymyxin B		X	NA	P	MAq	Current change	[95]
PB_52_-PEO_29_-LA	4.1	<1.1	0.25	Hemolysin	e^−^	X	NA	P	MAq	Current change	[94]
PB_52_-PEO_29_-LA	4.1	<1.1	0.25	Polymyxin B		X	NA	P	MAq	Current change	[95]
PB_92_-PEO_78_	8.4	1.08	0.34	AQP10	H_2_O	-	1:270	V	PFR & SE	Vesicle size change	-
PB_125_-PEO_80_	8.9	<1.1	0.28	Alamethicin	Calcein	-	1:2-1:8	V	MAq	Cargo release → Color change	[96]
PHEMA_25_-PBMA_25_-PHEMA_25_	14.3	1.30	0.83	AqpZ		-	NA	P	MAq & biobeads	Current change	[97]
PHEMA_25_-PBMA_25_-PHEMA_25_	14.3	1.30	0.83	Hemolysin		X	NA	P	MAq	Current change	[97]
PHEMA_25_-PBMA_25_-PHEMA_25_	14.3	1.30	0.83	OmpF		-	1:70	P	MAq & biobeads	Current change	[97]
PEE_37_-PEO_40_	3.9	<1.1	0.39	Alamethicin	Calcein	X	1:2–1:8	V	MAq	Cargo release → Color change	[96]
PPO_34_-PGM_14_	6.5	1.30	0.66	Strepatividin-BSA		ND	1:5, 1:15, 1:50	V	PPFR		[98]
PI_93_-PEO_87_	10.2	1.00	0.31	FhuA	TMB	X	1:6700. 1:5,300,000	V	MOr, SI & SEC	Cargo → Encapsulated enzyme activity → Color change	[73]
PEO_136_-PIB_18_-PEO_136_	8.0	1.86	0.90	Cecropin A	Calcein	X	1:30	V	MAq & SEC	Cargo release → Color change	[99]
P4MVP_21_-PS_26_-P4MVP_21_	13.1	NA	0.80	PR		X	1:10	V	MAq & precipitation	Absorbance change in membrane protein	[100]
P4MVP_21_-PS_38_-P4MVP_21_	14.3	1.19	0.74	PR		X	1:10	V	MAq & precipitation	Absorbance change in membrane protein	[100]
P4MVP_29_-PS_42_-P4MVP_29_	18.7	NA	0.78	PR		X	1:10	V	MAq & precipitation	Absorbance change in membrane protein	[100]
P4MVP_22_-PB_28_-P4MVP_22_	15.0	NA	0.92	PR		ND	1:10	V	MAq & precipitation		[101]
P4MVP_22_-PB_28_-P4MVP_22_	15.0	NA	0.92	RC	e^−^	X	1:25	V	MAq & precipitation	Cargo → Reduction of MP → EPR signal	[102]
P4VP_22_-PB_28_-P4VP_22_	7.1	NA	0.82	PR		ND	1:10	V	MAq & precipitation		[101]
P4MVP_29_-PB_56_-P4MVP_29_	17.4	1.08	0.81	RC	e^−^	X	1:25	V	MAq & precipitation	Cargo → Reduction of MP → EPR signal	[102]
P4MVP_18_-PB_93_-P4MVP_18_	13.9	1.06	0.62	PR		ND	1:10	V	MAq & precipitation		[101]

**Figure 3:**
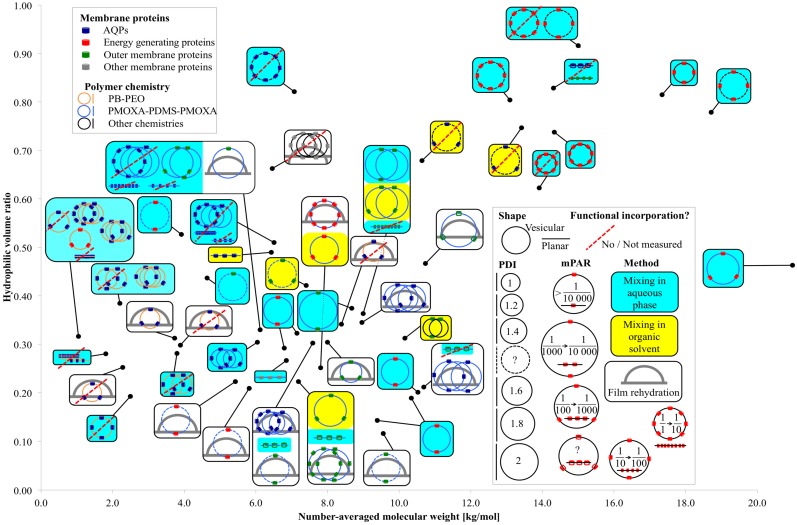
Overview of relevant parameters for membrane protein incorporation into amphiphilic block copolymers.

Figure 3 presents an overview of membrane protein incorporation into polymers with a known *M_n_* and *f*. Each black dot represents one polymer. The connected box shows the polymer chemistry, the incorporated membrane protein family, the self-assembled morphology (vesicular or planar), the incorporation method, the *PDI* of the polymer (not of the polymersomes), the mPAR and if the incorporation was functional or not, or respectively not measured. If there are several sketches in the box, several different experiments have been performed on the polymer. If there are two crossing circles and two close lines respectively, two different mPARs were investigated, where all other parameters remained the same. If there are three crossing circles, three or more mPARs were investigated. In the case of varying another parameter than mPAR (incorporation method, polymer chemistry, incorporated membrane protein *etc.*) a new sketch is drawn. Generally, polymers capable of functional incorporation have an *f* between 0.2 and 0.35 and *M_n_* was in between 2 and 12 kg/mol. Compared with PB-PEO, PMOXA-PDMS-PMOXA has a far broader *PDI* [46], its bilayer is highly water impermeable [17] and they do not collapse in dried form [47]. PB-PEO is more lipid-like as far it collapses easier and has higher water permeability [18]. The polymers that did not achieve functional AQP incorporation were mainly PB-PEO polymers with small *M_n_* and *PDI*. Energy generating (BR, CcO, NADH reductase, ATPase, RC, PR) and outer membrane proteins (OmpF, OmpG, FhuA, TsX) were incorporated mainly into PMOXA-PDMS-PMOXA polymers, but outer membrane proteins have also been incorporated in more exotic chemistries in an *f* range where one would not expect vesicular structures. The great majority of functional incorporation trials were performed with vesicular structures, where mixing was done in aqueous phase. Generally, at smaller *PDI* values, no functional membrane proteins can be incorporated, which is in agreement with the findings from Pata *et al.* [20]. A wide range of mPARs have been used with no optimal ratios detected. However, mPARs are based on the initial or nominal concentrations of membrane proteins and polymers and the final mPAR after incorporation may be different [48]. In the next section, we will discuss how to quantify membrane proteins (with focus on AQPs) after incorporation.

## 3. Evaluation of AQP Incorporation Characterization Methods

Detecting functional incorporation of AQPs is challenging, as the permeating solute is neutral water molecules. Protein mediated transport of neutral molecules (in particular at the single protein level) is harder to measure than transport of charged molecules (ion or protons) or a specific chemical reaction (e.g., ATPase enzyme activity). Although deuterated water labeling has been proposed for measurements via Raman spectroscopy [103], these type of measurements is complicated by the fact that water transport rate in the AQP channel is different for deuterated water molecules compared to that for normal water molecules [104].

A popular method for measuring functional incorporation is SFLS. In SFLS, proteopolymersomes are rapidly mixed with an osmotically active agent (NaCl or sucrose) in a defined volume. In the case of a hyperosmotic shock, proteopolymersomes will shrink, which will give rise to an increase in light scattering. With an increasing amount of incorporated AQPs, the shrinking rate will increase as well. This method is however strongly affected by the quality (size distribution) of the polymersomes, of the AQP concentration in the polymersome and the concentration of the osmolytes [56].

In principle, direct visual quantification can be achieved by FF-TEM, although FF-TEM will not reveal any functional information. In FF-TEM, proteopolymersomes are captured in their original shape by quick-freezing. The frozen sample is fractured, where the fracture plane is along the proteopolymersome bilayer, which is the weakest point of the whole system. The sample with incorporated AQPs (or the cavities, where AQPs were embedded in the bilayer) is then exposed to carbon/metal coating. The forming replica is removed from the thawed sample and AQPs/cavities can be observed on the replica as distinct spots on the proteopolymersomes.

Another method is FCS of fluorescently labelled AQP. In FCS time-dependent fluctuations of fluorescence intensities in a microscopic space, the so-called confocal volume, are monitored and subjected to an autocorrelation function. Dependent from the different diffusion time of the particles diffusing through the confocal volume, one can obtain the number of particles in the confocal volume within a given time interval. When proteoliposomes or proteopolymersomes are monitored, then solubilized to micelles and monitored again, the proteins-per-vesicle-ratio (mean number of membrane proteins incorporated in the bilayer of one vesicle) can be obtained by dividing the latter number by the first. It is assumed that micelles contain only one AQP, thus the micelle-per-vesicle ratio is equal to the proteins-per-vesicle-ratio. Further details to the theory are given in [48]. Alternatively, one can obtain the proteins-per-vesicle-ratio by correlating the proteopolymersome solution with the AQP stock solution. Both correlations have advantages and challenges that are further described in the FCS subsection.

In characterizing biological material, SAXS is also a versatile tool because it gives structural information on particles in solution on a length-scale from 1 to 100 nm where data are presented as scattering intensity as a function of the magnitude of the scattering vector *q*. This quantity is independent of the particular geometry of the experimental set-up and directly related to the scattering angle 2θ as *q* = 4π sin(θ)/λ where λ is the wavelength of the X-ray beam. Two scattering points separated by a distance *d* within a particle gives rise to interference showing up as increased intensity in the scattering curve at *q* = 2π/*d*. This means that large features are probed at low *q* while smaller details are probed in the high-*q* region of the curves. The strength, with which a particle scatters, its contrast, is proportional to its excess electron density, *i.e.*, the difference between the electron densities of the sample and the solvent. The downside is that SAXS requires access to elaborated synchrotron radiation sources.

Here, we will exemplify SFLS, FF-TEM, FCS and SAXS analyses with a series of diblock copolymers with optimal *M_n_* and *f* range for functional membrane protein incorporation: PB_29_-PEO_16_, PB_33_-PEO_18_, PB_45_-PEO_14_, PB_43_-PEO_32_, PB_46_-PEO_32_ and PB_92_-PEO_78_. PB-PEO was chosen because it showed functional AQP incorporation as discussed before and the *Mn* and *f* range is easier to control compared to PMOXA-PDMS-PMOXA. For SFLS, FF-TEM and SAXS, AqpZ is used as the incorporated membrane protein, where GFP-tagged human aquaglyceroporin AQP10 is used for the FCS experiments. Details are provided in the supporting material.

### 3.1. Stopped-Flow Light Scattering

**Figure 4:**
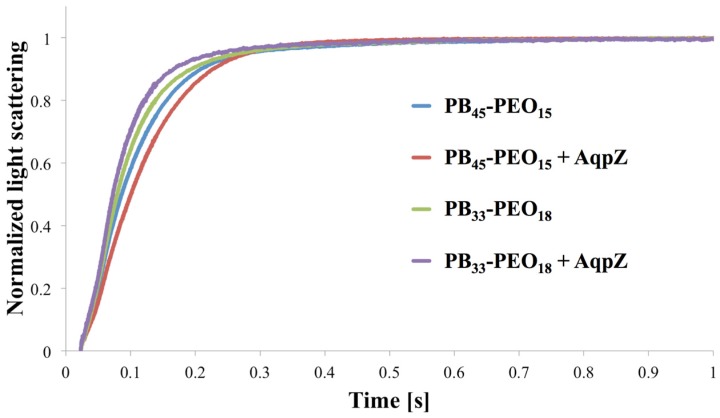
Normalized light scattering *vs.* time for proteo- and polymersomes of PB_45_-PEO_14_ and PB_33_-PEO_18_ at an mPAR of 1:100. For PB_45_-PEO_14_ the apparent water permeability is slightly decreased for the proteopolymersomes *versus* polymersomes, whereas for PB_33_-PEO_18_ it is slightly increased.

In order to exemplify an SFLS analysis, data for PB_45_-PEO_14_ and PB_33_-PEO_18_ diblock copolymer proteo- and polymersomes (meaning with and without AqpZ) is shown in Figure 4. For PB_33_-PEO_18_ the rate constant associated with the increase in light scattering intensity was slightly higher with AqpZ, for PB_45_-PEO_14_ it was even lower. This illustrates one of the major challenges in SFLS. The absence of a significant response to the change in extravesicular osmolarity could be due to an increase in the bilayer bending modulus induced by the presence of (non-functional or blocked) ApqZ. We observed similar problems in previous experiments with AqpZ and SoPIP2; 1, where only the smallest polymers (PB_12_-PEO_10_ and PB_22_-PEO_23_) showed a significant difference in SFLS between proteo- and polymersomes (results not shown). Another reason for the similar SFLS signal might be the blockage of the AqpZ channels by PEO chains. In this case, AqpZ would simply sit in the bilayer as an impermeable hydrophobic block, as suggested from Kumar *et al.* [18], because water permeation is blocked by the areas corresponding to the incorporated AqpZ, lower permeabilities of proteopolymersomes can be expected as compared to polymersomes. On the other hand the incorporated AqpZ could be fully functional, but the polymer matrix is resistant to changes in volume. This underscores the notion that SFLS is not a stand-alone technique.

### 3.2. Freeze Fracture Transmission Electron Microscopy

**Figure 5:**
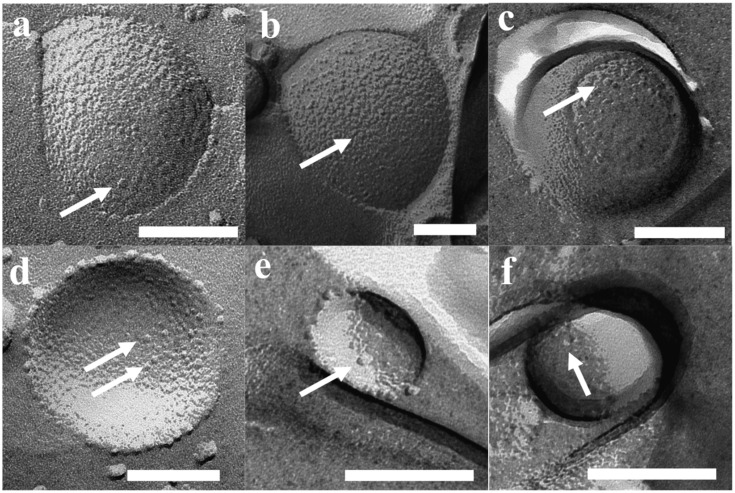
FF-TEM images of PB_45_-PEO_14_ proteo—(**b**,**c**,**e**,**f**) and polymersomes (**a**,**d**). All vesicles revealed spots, potentially not from AqpZ but rather collapsed PB chains (**a**–**c**) or bad fracturing artifacts (**d**–**f**). Scale bar is 100 nm.

Results of FF-TEM for PB_45_-PEO_14_ proteopolymersomes are shown in Figure 5. Proteopolymersomes with an mPAR of 1:100 were produced using film rehydration (FR), frozen and fractured in a Leica MED20 station, where two planchets with frozen sample are separated, thus the fracture is more a “crack” than a “cut,” thereby minimizing smearing effects from usual FF procedures (for details see supporting information). All proteo- and polymersome had a pronounced raspberry-like surface, potentially due to collapsed PB chains. However, the “typical” spots that have been claimed to be associated with AQP in a study on proteoliposomes [105] were not observed. In Figure 5, the bubble-like spots are distributed equally among polymersomes (Figure 5a–d) and proteopolymersomes (b,c,e,f). The spots could be either PB chain accumulations (Figure 5a–c) or artifacts due to bad fracturing (d–f). Proteo- and polymersomes of other PB-PEO polymers at other *M_n_* and *f* showed similar behavior. It thus seems that FF-TEM sample preparation plays a major role in false positive results. Occasionally, we observed dots all over the sample that were clearly not AqpZ but potentially polymer micelles. These dots could be eliminated by omitting an up concentration step and by carefully controlling temperature, sample and cutting handling or metal coating parameters (the optimized protocol is given in the supporting information). Even among the polymers with the shortest PB chains (PB_32_-PEO_30_ and PB_45_-PEO_14_), we could not ascertain the presence of AqpZ. However, from these experiments alone we cannot exclude the possibility that AqpZ tetramers could be present as the hydrophilic PEO chains are still large compared to lipid head groups. Thus the AqpZ could be concealed in the PB core.

### 3.3. Fluorescence Correlation Spectroscopy

As both SFLS and FF-TEM present challenges as tools for evaluating protein incorporation into polymersomes, we also evaluated FCS as a novel method for getting quantitative information about AQP incorporation. This was inspired by a recent paper by Erbakan *et al.* describing various AqpZ isoforms, tagged with a fluorophore in proteoliposomes, where the protein-per-vesicle ratio was determined and further substantiated using SFLS [48]. Initially, we attempted to reproduce the proteoliposome experiments described in [48]. At an mPAR of 1:200, our measurements revealed a proteins-per-vesicle-ratio of 5.35, which was comparable to the ones obtained in Erbakan *et al.* (around 7.5). The difference could be due to the different AQP and tagged fluorophore used.

**Figure 6:**
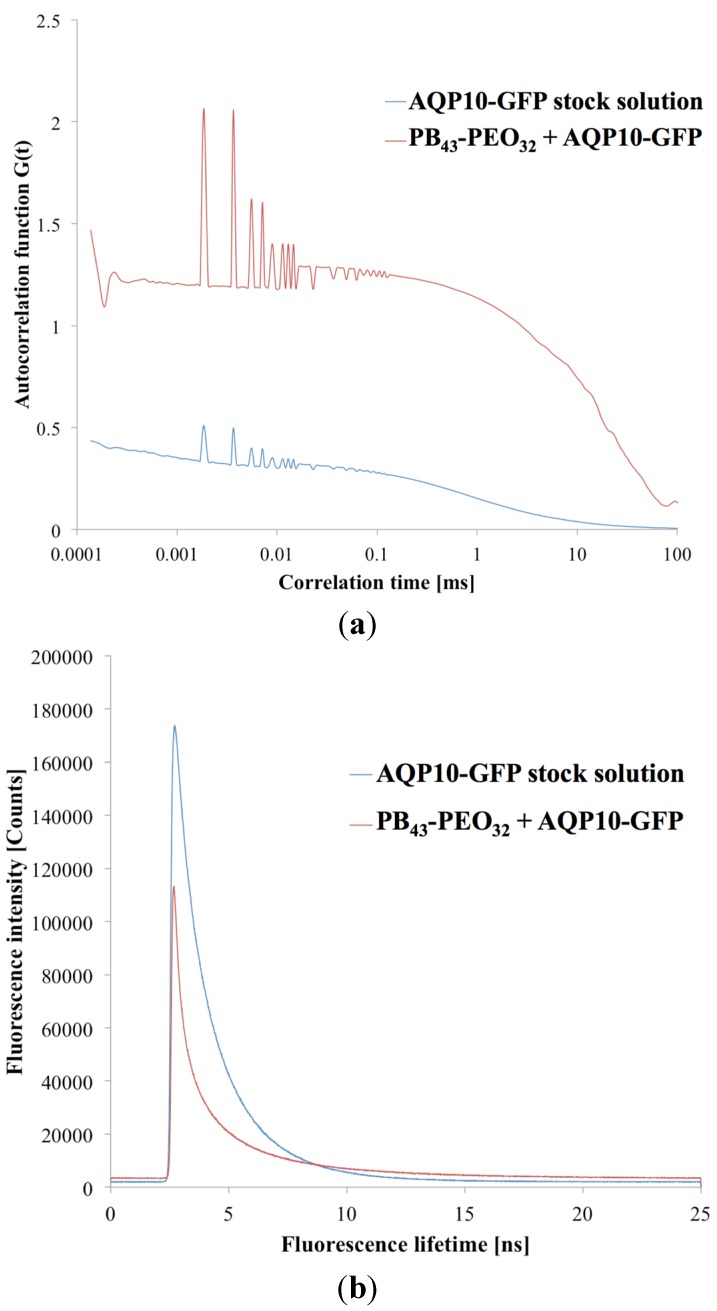
(**a**) Correlation diagram of proteopolymersomes and AQP10-GFP stock solution as a function of correlation time *τ* against autocorrelation function G(*τ*). The higher autocorrelation signal indicates a lower number of particles in the confocal volume, due to slower diffusion time. (**b**) Fluorescence lifetimes of the same samples as a function of lifetime against intensity signal. Where the intensities varied, the fluorescence lifetime was in a comparable range.

After having optimized the FCS instrument parameters for proteopolymersomes (for details please refer to the supporting information), we performed FCS on proteopolymersomes of PB_45_-PEO_14_ (mPAR 1:100) with AQP10-GFP and with OG-solubilized protein micelles. The results are shown in Figure 6. We obtained a higher species number in the proteopolymersomes sample than in the protein micelle sample. This could be due to the same OG-induced aggregation. We therefore decided to correlate the proteopolymersomes to the AQP10-GFP stock. Erbakan *et al.* could not do this, because the fluorophore used (mBanana fluorescent protein) exhibited a decreased fluorescence lifetime in pure OG environment (stock solution) compared to lipid/OG environment (solubilized protein micelles). GFP, however, did not seem to alter fluorescence lifetime significantly whether the AQP10-GFP is in OG (1.8 ns) or polymer/OG environment (1.97 ns, Figure 6b). They are comparable to fluorophore used by Erbakan *et al.* (4 ns [48]) and to standard GFP fluorescence lifetime (3 ns [106]). The difference between our GFP fluorescence lifetime and the standard one could be due to shielding of the attached AQP10 and the OG environment, as well as to the fitting algorithm of the instrument.

Accordingly, the sample correlation depend on the single components of the system. In the case of sensitive fluorophores, it is better to compare AQP vesicles and AQP micelles not to influence the fluorophore environment. In the case of polymers as the protein matrix, it is better to correlate the AQP-fluorophore stock solution as the polymeric AQP micelles aggregate easily. A disadvantage of correlating AQP-fluorophore stock with AQP vesicles is that the final concentration of AQP is not known, complicating a correlation with similar AQP concentration.

Calculating the species number of pure AQP10-GFP from the stock in the confocal volume and the one from the proteopolymersome solution (Figure 6a), we obtained a proteins-per-vesicle-ratio of 2.87. These results demonstrate that FCS can serve as a tool to quantify AQPs in proteopolymersomes. This opens possibility for conducting a systematic study in which *f* and *M_n_* are varied in order to obtain quantitative information about which polymers can be used to achieve the highest proteins-per-vesicle-ratio.

### 3.4. Small-Angle X-Ray Scattering

**Figure 7:**
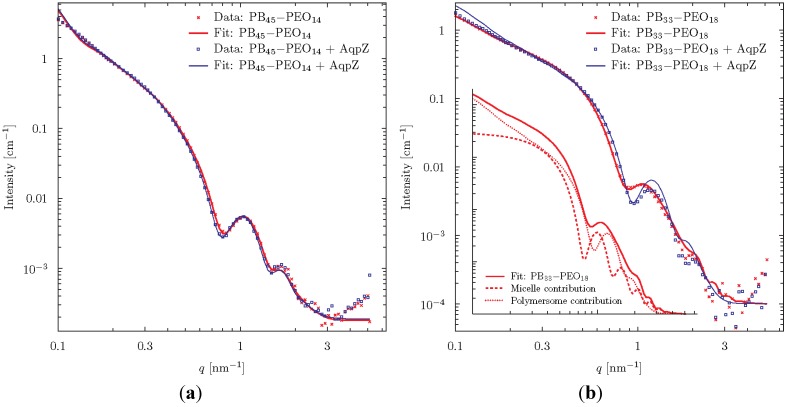
SAXS data for proteo- and polymersomes of PB_45_-PEO_14_ (**a**) and PB_33_-PEO_18_ (**b**). The fits were obtained using a vesicle model consisting of three concentric spherical shells. To fit the polymersomes of PB_33_-PEO_18_, it was necessary to include an additional contribution from block-copolymer micelles as shown in the insert.

Scattering curves for FR prepared proteo- and polymersomes of PB_45_-PEO_14_ and PB_33_-PEO_18_ are shown in Figure 7. The samples were extruded and centrifuged prior to measurements. At low *q*-values a typical linear slope is observed in the log-log plot, with the intensity following a power law of *q*^−2^. This behaviour is typical of flat laminar structures. The fact that the slope extends below the lowest detectable *q*-region indicates a low curvature (flat structure) even on the largest detectable length scale of *q* = 2π/0.1 nm ≈ 60 nm. At higher *q*, a characteristic oscillatory behaviour is observed. This is attributed to the complex interference between the negative contrast of PB and the positive contrast of PEO.

The theoretical scattering from various simple geometrical objects such as spheres, cylinders and ellipsoids of varying contrast can readily be calculated. These can be combined to form simplified models of the studied particles. We choose to analyze the data using a vesicle-model consisting of three concentric spherical shells of alternating contrast, corresponding to shells of PEO, PB, and PEO, respectively. The thickness of the individual shells was varied to give the best fit to data using a least squares fitting routine.

Excellent fits were obtained for the PB_45_-PEO_14_-system meaning that data are in good agreement with the assumption that the diblock copolymers form spherical vesicles. The fits were especially sensitive to changes in the parameter determining the thickness of the central hydrophobic bilayer constituted by the PB-groups. These were fitted to 9.10 ± 0.1 nm and 8.94 ± 0.07 nm in the presence or absence of AqpZ respectively. Concerning the overall vesicle diameter, we can conclude from the model that it is larger than 60 nm, which is not surprising given the initial analysis above. It is evident from the data and the fit parameter values that well defined bilayer vesicles are formed and that the incorporation of AqpZ introduces only minor differences to the structure of the vesicles.

For the PB_33_-PEO_18_ proteopolymersomes, reasonably good fits were obtained with the vesicle model with a hydrophobic bilayer thickness of 7.66 ± 0.05 nm. However, for the polymersomes, no fit to the data gave reasonable physical parameters. The data fit required the assumption that a population of block copolymer micelles co-exists with the vesicles. The combined model fit showed that 76 wt% of the population consisted of proteopolymersomes and 24 wt% were micelles with a hydrophobic core of diameter 11.7 ± 0.3 nm gives a good fit with the data. The insert of Figure 7 shows the separate vesicle and micelle contributions.

In conclusion, the SAXS analysis reveals that, for PB_45_-PEO_14_, vesicles are formed both with and without AQP where AQP incorporation leads to a minor differences in average hydrophobic vesicle wall thickness, which could indicate a dimpling or puckering of polymers close to the incorporated AQPs. In the case of PB_33_-PEO_18_, some micelle-formation is observed, but this tendency is reduced when AQP is incorporated.

To summarize this chapter, the characterization methods for functional incorporation of AQPs in PB-PEO diblock copolymers were investigated. SFLS and FF-TEM are in principle powerful tools but, for polymer systems, the analysis can give ambiguous results. On the other hand, FCS and SAXS can provide detailed information, but the latter requires access to large-scale facilities in the form of synchrotron radiation sources.

## 4. Recent Developments in AQP Membrane Designs

Provided that the performance of AqpZ proteopolymersome described by Kumar *et al.* [17] could be scaled up, they could create a water separation membrane that reaches fluxes of 11,000 L m^−2^ h^−1^, a value that is several orders of magnitude higher than conventional industrial membranes. In highly packed 2D AqpZ crystal arrays, fluxes of up to 16,000 L m^−2^ h^−1^ could be achieved in principle [107]. However, these values will very probably never be achieved due to upscaling issues—but they show the huge potential of biomimetic membranes. The development is rapid: in 2011, ABPMs were regarded as the most revolutionary membrane advances but also the ones most farthest away from a potential commercial viability [108]. Now, four years later, ABPM membranes are commercially available with areas of tens of m^2^ [109]. It will still take time before the technology is widespread, but it has definitely moved outside the fundamental research domain. In the next sections, we will highlight the AQP biomimetic membrane technology development in detail.

### 4.1. Membrane Designs Based on Planar Biomimetic Structures

The first industrial approaches are made from two Danish companies, Aquaporin A/S, and AquaZ (now Applied Biomimetic). Together with the Danish Technical University (DTU), the University of Southern Denmark (SDU), DHI, Lund University, Sweden, Ben-Gurion University of the Negev, Israel, Malaga University, Spain, Vilnius University, Lithuania and Veolia Water, France, Aquaporin A/S joined the EU funded MEMBAQ project 2006–2010 which aimed at utilizing AQPs for industrial applications [110]. At the same time, AquaZ started on membrane development based on a patent from Carlo Montemagno where he described conceptually how AQPs, embedded in polymeric or lipid bilayers, could function as a biomimetic membrane, although without any concrete design of such a membrane [111].

The first membrane design from Aquaporin A/S was based on an ethylene tetrafluoroethylene (ETFE) scaffold with holes of 300 µm, produced by laser-ablation, which are inspired by painting/folding lipid chambers from the 70s [112,113]. A freestanding lipid-bilayer film is established by “painting” a two-phase solution over the hole, where the lipids move from the organic solvent to the aqueous phase, accumulate around the holes and establish a bridging layer. Several membrane proteins and peptides were incorporated in the freestanding layer including porins [36]. In addition, freestanding PMOXA-PDMS-PMOXA polymer membranes with incorporated gramicidin A channels were developed [37] and characterized [114]. In subsequent designs, the membrane is supported by PEO-dimethacrylate (PEO-DMA) based hydrogels [115] or stabilized using surface plasma polymerization [116]. Moreover, a strategy was explored to form interface lipid bilayer between lipid-coated water drops in a continuous oil phase [117]. A later liquid membrane approach investigated SoPIP2;1 proteoliposomes in a sandwich between NF membranes that could prove an AQP fingerprint for the first time, however at modest water flux [118]. These designs [118,119,120,121,122] later paved the way for developing membrane-based biosensor designs [45]. The hydrogel approach from Aquaporin A/S was adapted in 2010, when Montemagno and AquaZ claimed an ABLM design with internally UV cross linked and PA-interconnected proteoliposomes that are immobilized on a lipid-coated PA layer and supported with a PEO hydrogel [123].

In 2009, Aquaporin A/S and DHI Singapore initiated collaborative research with the SMTC on biomimetic membranes. At the same time, the Chung lab from NUS started biomimetic research in collaboration with Wolfgang Meier and coworkers. NUS followed up on Aquaporin’s hydrogel approach and tried to achieve a planar proteobilayer, starting with AqpZ proteoliposome fusion on pure and PEO coated porous alumina and found an increasing stability with increasing mPAR [124]. In 2012, they described an approach based on a Langmuir-Blodgett-film with Nickel-chelated lipids that bind to His-tagged AqpZ, similar to the approach from Kumar [29] but using lipids with subsequent Langmuir-Schäffer deposition-mediated transfer on a mica surface [125]. This was followed by Kaufman *et al.* who incoporated spinach AQP (SoPIP2;1) in positively charged bolalipid micelles which were then fused on a negatively charged silica surface [126]. Chuyang Tang *et al.* investigated on fusion behavior of proteoliposoes on pure and polymer-coated silica via quartz crystal microbalance with dissipation (QCM-D) [127]. They found increasing robustness and fusion resistance with increasing mPAR, and further proteoliposome stabilization with polyelectrolyte layers at the highest mPAR (1:25) in 1,2-diphytanoyl-sn-glycero-3-phosphocholine (DPhPC) liposomes [127].

The SMTC group also investigated ABLMs, following Kaufman’s approach of liposome fusion on nanofiltration (NF) membranes [128,129] and fused AqpZ proteoliposomes on NF PA-polysulfone (PSf) membranes that were precoated with positively charged lipids via spin-coating [130]. The proteoliposomes were placed on the NF membrane and slightly pressurized with 0.5 bar. They found a linear relationship between the roughness of the ABLM surface and mPAR indicating AqpZ incorporation, but no effect from AqpZ on the water flux *J_v_* and the reverse salt flux *J_s_* could be observed [130].

### 4.2. Membrane Designs Based on Vesicular Biomimetic Structures

A different approach was initiated jointly by SMTC and Aquaporin A/S in which AqpZ proteoliposomes were embedded in the standard PA layer made from interfacial polymerization of *m*-phenyl diamine (MPD) and trimesoyl chloride (TMC) on a PSf support structure [1,131,132]. ABLMs were tested with functional AqpZ proteoliposomes, proteoliposomes with an inactive AqpZ mutant and PA-PSf membranes without proteoliposomes. ABLMs were further benchmarked against commercially available membranes with cross-flow RO tests on 42 cm^2^ effective coupon area. The ABLMs with AqpZ proteoliposomes had a significantly higher *J_v_* than the ABLM with inactive AqpZ and the PA-PSf membrane while *J_s_* values were similar in all cases. Furthermore the ABLMs were able to withstand 10 bar pressure making them well-suited for low pressure RO applications. *J_v_* of the AqpZ ABLM was ~40% higher compared to the commercial brackish water RO membrane (BW30) and an order of magnitude higher compared to a seawater RO membrane (SW30HR).

This was followed up by a systematic study, which revealed that 1,2-dioleoyl-sn-glycero-3-phosphocholine (DOPC)-based proteoliposomes and proteoliposomes of mPAR of 1:200 gave optimal water flux as judged by SFLS and that cholesterol addition could seal defects on the proteoliposomes [133].

To achieve higher loading and better sealing, the SMTC group coated proteoliposomes with polydopamine (PDA) and immobilized them on a 28 cm^2^ NF polyamide imide (PAI) membrane by embedding them in branched polyethyleneimine (PEI), cross linked per PA bond at elevated temperature [134]. The SFLS data showed, that the elevated temperature had a higher negative influence on the permeability of the proteoliposomes than the PDA coating itself. Even so, AqpZ function was demonstrated with an optimal performance mPAR of 1:200 when reconstituted and integrated into the PAI-PEI layer. In contrast, the best SFLS response was achieved at an mPAR of 1:100 [133]. This discrepancy could be due to AqpZ being affected by the PDA coating or the PEI branches. Still, the *J_v_* was measured to be 36 L m^−2^ h^−1^ bar^−1^) making it the highest among all biomimetic membranes so far [134].

In addition, proteopolymersomes can be functionalized to get bound chemically to a counterpart functionalized membrane. Functionalization of both liposomes and polymersomes has been studied extensively since decades [135,136,137].

ABPMs with functionalized proteopolymersomes are first mentioned in a patent of Montemagno in 2011, where he claimed a concept of proteopolymersomes made of polyethyloxazoline-polydimethylsiloxane-polyethyloxazoline (PEOXA-PDMS-PEOXA) triblock copolymers, where the methacrylate-functionalized PEOXA block is immobilizing the proteopolymersomes on a methacrylate functionalized cellulosic membrane [138].

The first experimental results on this approach were presented by the NUS group [53]. They made proteopolymersomes containing AqpZ in methacrylate-functionalized PMOXA-PDMS-PMOXA and tested them with SFLS. In contrast to Kumar [17], no significant difference SFLS signals with varying mPAR was observed—likely a reflection of the issues with SFLS on rigid structures mentioned in Section 3. Proteopolymersomes were deposited onto acrylate-functionalized polycarbonate track-etched (PCTE) membranes and immobilized by UV-crosslinking of the acrylate groups with the methacrylate of the PMOXA, as claimed from Montemagno *et al.* [138]. Afterwards, the proteopolymersomes were further immobilized by pressure-assisted adsorption and possibly ruptured by “smooth extrusion”. AQP resulted in an increasing *J_v_* with increasing mPAR, whereas there was no *J_v_* with polymersomes alone; however, AFM and field emission-scanning electron microscopy (FE-SEM) revealed that the layer had some defects [53]. In a subsequent study, they followed the same approach using an acrylate-functionalized cellulose acetate membrane [54]. Here, they found an increase in *J_v_* and decrease in NaCl rejection with proteopolymersomes of higher mPAR. The increase in *J_v_* could indicate AQP activity, but the NaCl rejection was however still quite low (33%) and the measured membrane area was only 7 mm^2^ [54].

In another approach, gold-disulphide binding to immobilize disulphide functionalized PMOXA-PDMS-PMOXA AqpZ proteopolymersomes on gold-coated porous alumina and silicon surfaces has been described [77]. Here, FE-SEM revealed that full coverage of the pores was achieved at pore diameter of 55 nm, where larger (100nm diameter) pores remained open. Again, an effect of incorporating AQP was observed but NaCl rejection was modest [77]. To obtain a better sealing, cysteamine was added with PDA and histidine coatings after the proteopolymersome immobilization on gold-coated PCTE [3]. *J_v_* increased and *J_s_* decreased with increasing amount of PDA-His-layers; however, the best sealing was obtained without proteopolymersomes. Pressure retarded osmosis (PRO) mode testing (AL to the water receiving draw side) resulted in significantly higher *J_s_* than forward osmosis (FO) mode testing (AL to feed side) [3]. Mathematical simulations on this ABPM indicated that in PRO mode, *J_v_* is determined by vesicle size and permeability. In FO mode, hydrostatic pressure is determined by the vesicle interior solute concentration [52].

Another slightly different design has been experimentally realized afterwards with AqpZ and methacrylate- and carboxyl-functionalized PMOXA-PDMS-PMOXA on amine-functionalized CA [63]. Here, proteopolymersomes are first covalently attached to the CA, where the carboxyl-groups of PMOXA and the amine groups on CA formed a PA bond. Then, a methacrylic cross linking polymerization is performed by dipping the membrane into a mixture of methyl methacrylate, ethylene glycol dimethacrylate and initiator. *J_v_* is linearly increasing and NaCl rejection decreasing with polymerization time. An increase in *J_v_* and decrease in NaCl rejection of ABPMs compared to only methacrylated CA and polymersome coated CA in both FO and NF mode evidenced the presence of AQP. However the NaCl rejection (61%) still indicated significant defects [63].

Another example of methacrylate cross-linking involves amine-functionalized AqpZ proteoliposomes on a PDA precoated ultrafiltration (UF) polyacrylonitrile (PAN) membrane [139]. Here, proteoliposomes are internally cross linked via methacrylate and gently pressurized onto the PDA-PAN support, allowing the amines of PDA and functionalized lipids to react. Further stabilization is achieved via glutaraldehyde. The internal cross linking of the proteoliposomes has a positive effect on stability. *J_v_* and NaCl rejection between liposome-coated membranes and ABLMs showed some effects of AqpZ presence; however, FE-SEM images and low NaCl rejections revealed that defects in the ABLM played a strong role in the membrane performance [139].

Instead of chemical bonding, proteoliposomes or -polymersomes can be bound by electrostatic forces. Using this approach, Kaufman *et al.* attempted to fuse positively charged bolamphiphilic proteoliposomes onto negatively charged NF PA and sulfonate PSf (PSS) membranes [140]. The proteoliposome loading was enhanced with the more negatively charged PSS membrane. However, proteoliposome loading also led to a decrease in *J_v_* together with an increase in NaCl rejection, probably due to induced defects in the bolamphiphilic bilayer by SoPIP2;1 [140].

Another electrostatic-binding-based approach employed the embedment of positively charged poly-L-lysine covered AqpZ proteoliposomes in the anionic part of a layer-by-layer (LbL) sandwich on an UF PAN membrane [2]. The anionic part is made of polyacrylic acid (PAA) and PSS, where the cationic counterpart was polyallylamine hydrochloride (PAH). Here, a clear AqpZ effect could be observed as *J_v_* increased by 30%–50% after addition of proteoliposomes, where the effect was stronger when there was a higher amount of negatively charged lipids present. The MgCl_2_ rejection was comparable to the work of Zhao *et al.* [1]; however, no NaCl rejection was presented [2]. This work was extended by encapsulating magnetic nanoparticles to force more proteoliposomes magnetically to adsorb on the polyanionic film. In FO mode, they measured an increase in both, *J_v_* and *J_s_* with increasing mPAR, which speaks for remaining defects despite the efforts to load more vesicles onto the supporting substrate [141].

Wang and coworkers from Ocean University of China followed up on that approach and immobilized AqpZ proteoliposomes with positively charged lipids on top of a negatively charged PSS layer, followed by PEI on an UF PAN membrane [142]. Modest NaCl rejection and *J_v_* decrease indicated a highly defective membrane. An increase in *J_v_* between liposomes and proteoliposomes as well as further increase in *J_v_* with higher mPAR could indicate the presence of AQP. NaCl rejection remained however unchanged between all membranes. They further showed that membrane performance was compromised after detergent treatment [142].

**Figure 8:**
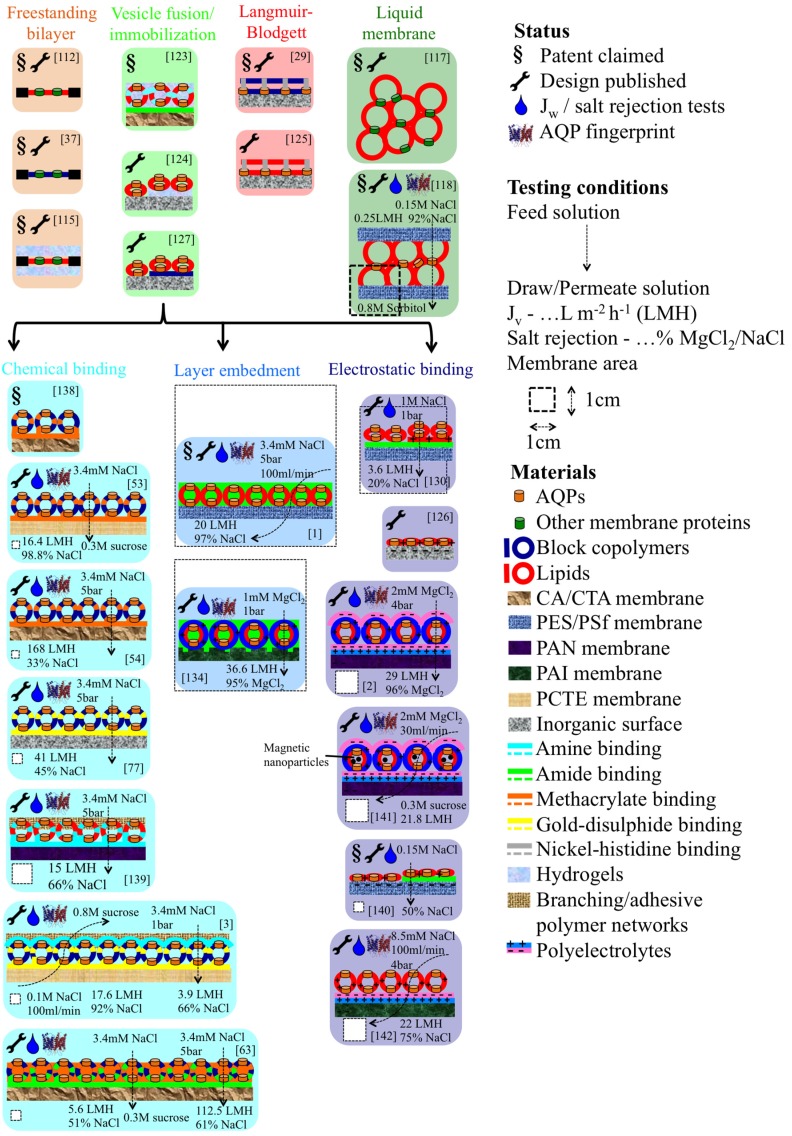
Schematic overview of all published designs for ABPMs and ABLMs. Pioneer work is mainly done by Kumar and Aquaporin AIS. The most experimental designs has been done by NUS, where NTU published the most promising layer embedment ABLMs. The main recent work is on LbL-based electrostatic binding, for example binding of proteoliposomes on a polyelectrolyte layer [142].

All designs are summarized in Figure 8, and based on the results obtained so far, we conclude that the embedment of proteopolymersomes or -liposomes in a layer results in more efficient membranes than layer-based immobilization. A great advantage of the PA-embedment technique is that no precoating/functionalization is needed which otherwise severely limits any upscaling [1]. All reported performances are however still modest compared to theoretical predictions and clearly more development is required. The major dilemma seems to be that with increasing mPAR, *J_v_* increases, but the matrix layer becomes weaker and more prone to salt leakage. Introduction of sealing and stabilizing polymer networks could improve rejection but may also compromise *J_v_* [107].

Next to ABPMs and ABLMs, there are two main directions aiming to achieve biomimetic membranes by AQP mimicking artificial channels: carbon nanotubes (CNTs) and organic building block nano channels [143]. CNT is more prominent because fast water permeation is proven in theory [144] and experimentally [145]. With regard to organic nano channels, there are five promising structures found to compete with ABPMs, ABLMs and CNTs: zinc and N,N-diacetic acid imidazolium bromide based zwitterionic coordination polymers [146], helical pores of dendritic dipeptides [147], imidazole compounds with urea ribbons [148], hydrazine-appended pillar[5]arenes, macrocycles of m-phenylene ethynylene [149]. Their great advantage is their smaller size with comparable channel diameter (3–10 Å) [143].

### 4.3. POSS—A Novel Element in Interfacial Polymerization

Nearly all RO and FO membranes are PA-based, often referred to as thin film composite (TFC) membranes due to their superior performance compared to other membrane designs. A PA-layer is generally generated by a reaction between an amine and an acyl chloride [150]. This reaction can be prepared by dissolving the amine group in an aqueous phase, and the acyl chloride group in an organic phase [151]. Typically, a membrane is wetted with the aqueous phase, containing the amine group and dried a little to remove visible liquid while keeping the surface moist. Then the organic phase with the acyl chloride group is added on top. The reaction growth is believed to be directed into the organic phase [152], due to a preferential solubility of the amine group in the organic phase compared to the solubility of the acyl chloride in the aqueous phase. This results in the well-known ridge and valley form of PA layer. The standard amine-acyl chloride combination is MPD and TMC, and typically these are supplemented with additives (molecules with similar chemistries) in low concentrations to improve flux, rejection or chlorine resistance [150].

The ideal AL of a PA membrane for water separation has to be highly water permeable, while rejecting all other solutes and being resistant against cleaning. An ideal AL of an ABPM could even be water impermeable if it enables sufficient integration of proteopolymersomes in such a way that water only passes the incorporated proteins. Therefore, novel AL components have to be explored. An AL with homogenous thickness could facilitate such proteopolymersome integration.

**Figure 9:**
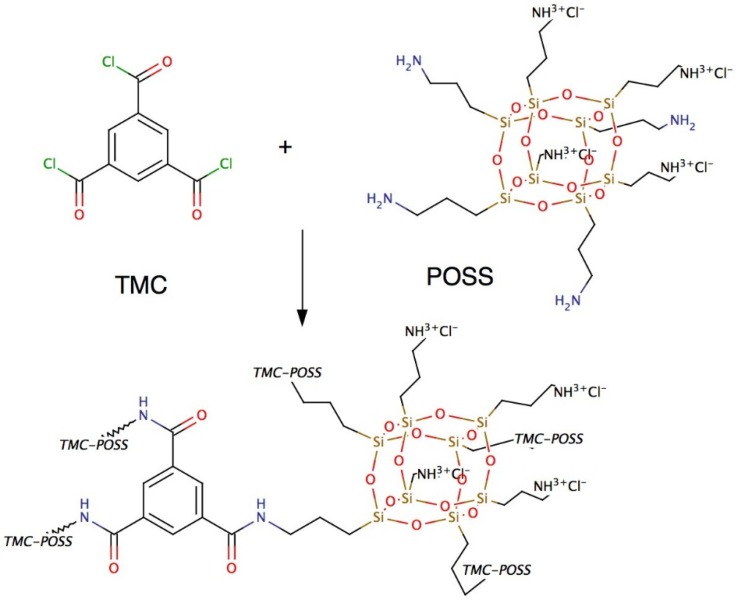
Chemical structure of POSS and TMC and the resulting AL. POSS as the amine linker generate a highly stable and well-defined AL with TMC.

We have used POSS (amine linker) and TMC (acyl chloride linker) for their potential use for the integration of proteopolymersomes in ABPMs. In a recent study POSS has been introduced as an AL layer components and POSS-TMC-layer exhibited a well-defined layer without ridges and valleys but with high mechanical stability on PAN membranes [153]. This may be a better platform for the integration of proteopolymersomes compared to the ridge-and-valley MPD-TMC network. The reaction is schematically depicted in Figure 9.

Here, we prepared PA layers (hereinafter referred to as AL) of POSS+TMC containing polymersomes of PB_29_-PEO_16_ in the aqueous phase. The influence of vesicles on the AL properties were determined, in order to provide a basis for subsequent addition of AQPs. We selected PB_29_-PEO_16_ due to its ability to form large amounts of stable polymersomes in aqueous phase compared to other PB-PEO polymersomes [154]. For the microfluidic approach, we used proteopolymersomes (AqpZ, PB_33_-PEO_18_, mPAR 1:100). PB_33_-PEO_18_ forms large amounts of stable polymersomes in aqueous phase as well and showed successful AqpZ incorporation as evidenced by SAXS. We used MilliQ water as the aqueous phase and hexane as organic phase and in order to achieve the lowest possible polydispersity, polymersomes were sonicated resulting in 95% of the polymersomes with a diameter of 196±83 nm as determined by dynamic light scattering (DLS).

We produced a non-supported AL by simply adding both phases after another in a beaker and an AL supported by microfiltration (MF) polyethersulfone (PES) layers using different coating procedures (for details see supplementary information). Characterization of the non-supported AL was done using Fourier-transformed infrared spectroscopy (FTIR), SEM and a novel, recently published microfluidic approach that allows for direct monitoring of the polymerization process [10]. Characterization of the supported AL was also achieved via FTIR and SEM, where it was also tested for functionality using standard flux and rejection test in FO mode and methylviolet staining.

**Figure 10:**
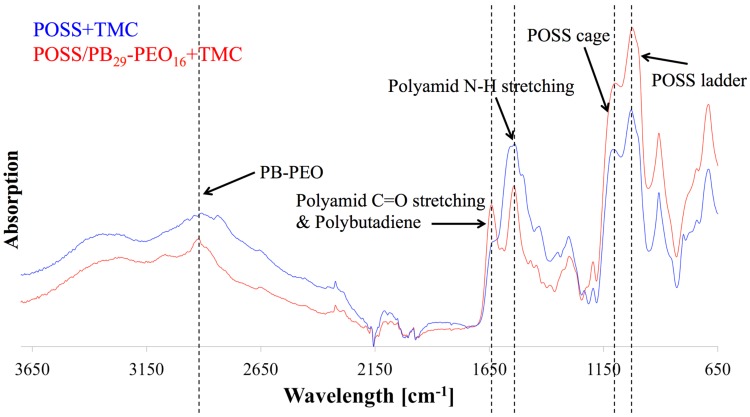
FTIR diagram of POSS/polymersomes+TMC AL (labelled red) and POSS+TMC control AL (labelled blue) as a function of wavelength against absorption. The AL with polymersomes had an absorption peak around 3000 cm^−1^, that responds to PB and PEO, indicating their presence in the AL, where the characteristic absorption peaks for PA bonds and POSS were present as well.

After adding both phases, pieces of the formed (non-supported) AL were air dried then vacuum-dried where they crumbled to flake-like structures. FTIR analysis of POSS+TMC with addition of polymersomes revealed the presence of block copolymers in the AL, see Figure 10. AL with polymersomes had an absorption peak around 3000 cm^−1^ (C-H stretch), which can also be found in spectra of PB and PEO [155,156]. The polymersome-free AL exhibited a broad peak at that wavelength range but not a distinct maximum as for the polymersome-containing AL. This could indicate a successful polymersome integration in the AL. Polymersomes furthermore did not seem to block PA formation, because the characteristic peaks of a PA bond, the C=O stretch at 1636 cm^−1^, as well as the N-H stretch at 1545 cm^−1^ [153] were clearly visible in the AL with polymersomes. Finally, partial hydrolysis of the POSS leading to the AL formation is not substantially affected by the presence of the polymerosomes as far as the characteristic peaks for the POSS-cage and ladder (1125 cm^−1^ and 1040 cm^−1^ [153]) were present in both AL. There was however an apparent influence of the polymersomes on TMC reactivity. Originally, Dalwani *et al.* used 2g/L TMC for their non-supported and supported AL [153]. In our case we could not form a non-supported AL with 2 g/L but with 0.5 g/L TMC. Potentially the TMC-POSS-stochiometry was artificially increased by the presence of another species in the aqueous phase. An excess of TMC could hinder network structure formation, because TMC will not connect POSS cages, resulting only in low molecular weight networks. We used 0.5 g/L TMC for the non-supported POSS+TMC AL and POSS/polymersomes+TMC AL.

**Figure 11:**
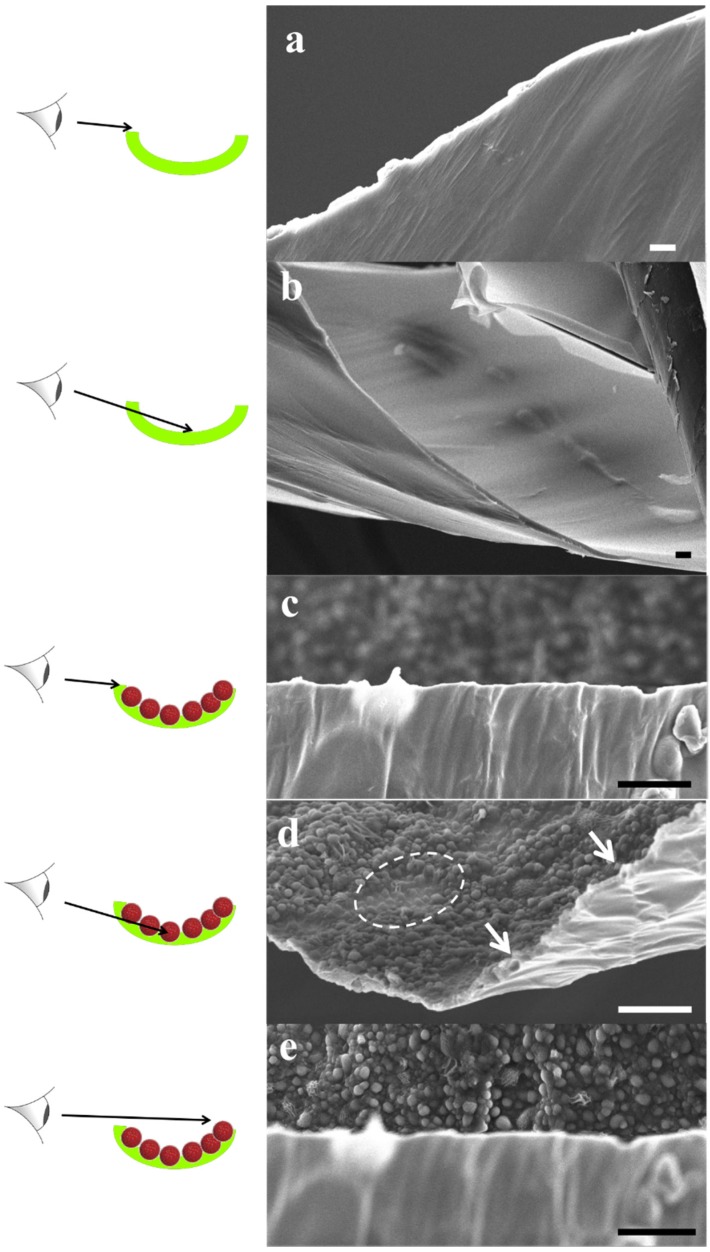
SEM images of POSS+TMC AL (**a**,**b**) and of POSS/polymersomes+TMC AL (**c**–**e**) with schematic sketches, which part of the layer is being captured. Images were taken from different parts of the flakes (labelled green in the sketch) of the AL, that were generated during the SEM preparation. The AL without polymersomes was smooth and well-defined, which remained on the organic side when polymersomes were added. The aqueous side was covered with loosely attached and half-covered polymersomes (dotted circle in (**d**)). A few could be observed inside the AL (arrows in (**d**)). Scale bar is 3 µm.

The FTIR results were complemented with SEM analysis of the same samples, see in Figure 11. The POSS+TMC AL appeared smooth and well-defined, in agreement with previous work [153], see Figure 11a,b. When polymersomes were added (Figure 11c–e) a clear distinction can be made between the side towards the organic phase, that does not reveal presence of polymersomes (Figure 11c) and the side that faced the aqueous layer, which is well-covered with polymersomes (Figure 11e).

Most of the polymersomes seemed to sit loosely on top of the AL, whereas some polymersomes seemed to be covered to a certain extent by the AL, their shape less sharp than the others (indicated by the dotted circles in image Figure 11d). A few polymersomes were directly embedded inside the AL, visible from its cracked profile (arrows in Figure 11d). This could indicate that the POSS approach can be used to embed polymersomes in such a way that they would be suitable for membrane fabrication.

Recently, a novel approach from the microfluidic field was published [10] that allows visual study of the evolution of the location of interfacial polymerisation reactions. This involves a chip containing a hydrophobized micro-chamber that is separated in two compartments by an array of micro-pillars each with a diameter of 30 µm and a height of 50 µm. The aqueous phase with amine linker was introduced via micro capillary connections into one compartment and formed a water-air-interface between the pillars. Then the organic phase with acyl chloride linker was introduced into the other compartment. AL formation at the interface between the solutions was observed using an optical microscope. Depending on the linkers, the resulting AL will have a different morphology and formation time. POSS+TMC forms well-defined AL with a formation time within 4 s. In contrast, for instance the apparent growth of a film from Jeffamine+TMC is not finalized after 15 min and the film reveals the ridge and valley structures that are typical for AL formed by interfacial polymerization [10].

**Figure 12:**
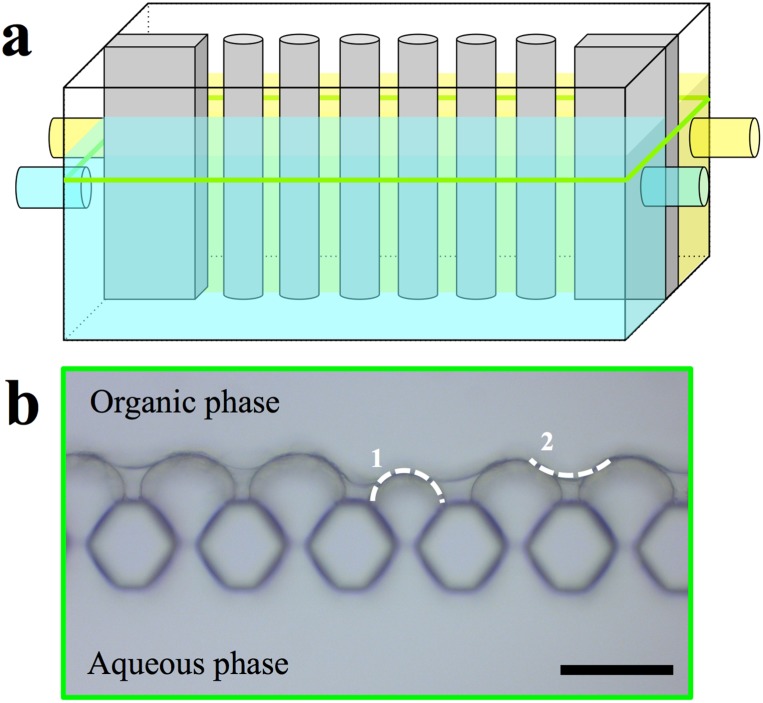
(**a**) Schematic sketch of the microfluidic chamber and micrographs of POSS/proteopolymersomes+TMC AL and (**b**) micrograph of the compartment. The aqueous phase reached into the other compartment. After introducing the organic phase, a well-defined AL formed. Scale bar is 50 µm.

We used this approach to monitor POSS/proteopolymersomes+TMC AL (AqpZ & PB_33_-PEO_18_, mPAR 1:100), see Figure 12. The chip that was used was not hydrophobized optimally, which resulted in partial infusion of the aqueous phase into the channel with the organic phase. The hydrophobization was still sufficiently efficient to hinder the aqueous phase from passing entirely to the other compartment. Other reasons for the shift of the interface from the pillar structures to the organic phase could be overpressure from the aqueous phase, which is hard to control since the offered pressure is in the range of 10^4^ Pa. When the organic phase containing TMC was introduced, the typical sharp AL was formed at the aqueous-organic interface (Figure 12b dotted line 1). After that, the reaction was continued by the diffused amine into the organic phase and the formed AL that connected the initial interfaces, exhibited a new aqueous-organic interface (Figure 12b dotted line 2). Such observation demonstrates a less denser AL formed by POSS/proteopolymersomes-TMC compared to that formed by POSS-TMC reaction. The formation time was on the order of seconds. The film remained in the same shape and no further growth was observed in the following 12 h.

**Figure 13:**
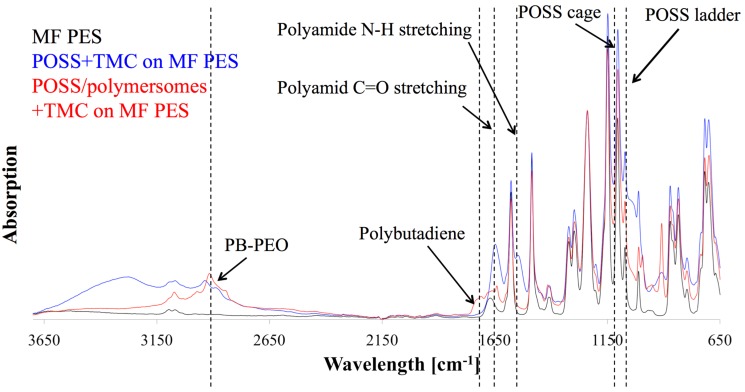
FTIR analysis of supported POSS/polymersomes+TMC AL (**red**) and POSS+TMC control AL (**blue**) on MF PES and pure MF PES (**black**). The PES supporting material had high absorption and interfered with many absorption peaks. A subtraction from the absorption spectra of pure PES resulted in negative peaks. We therefore only normalized the spectra. PB-PEO was present in the AL with polymersomes; however, the PA formation was strongly suppressed.

We then investigated POSS+TMC on MF PES support material coated following the procedures in Dalwani *et al.* [153], which is further described in the supporting information. The MF PES itself was supported by a nonwoven. FTIR spectroscopy revealed the presence of polymersomes in the supported AL, however, the PA formation is significantly reduced compared to the non-supported POSS/polymersomes+TMC AL, see Figure 13. A main challenge of analyzing supported AL with FTIR is the potential absorption of the PES support, which has a strong absorption especially in the region between 700 and 2000 cm^−1^. Especially, the POSS absorption peaks interfered strongly with PES peaks. In the supported POSS/polymersomes+TMC AL the PB-PEO signal at 3000 cm^−1^ was present as well as another small peak around 1700 cm^−1^ that also appeared in the FTIR spectra of PB [155]. Interestingly, it could not be found in the non-supported AL. Potentially, it was overlayed from the background signal in the region between 1600–3650 cm^−1^ that was more significant at the FTIR analysis spectra from the non-supported AL. Both PA bonds were present in the supported POSS+TMC AL but strongly reduced in the one with polymersomes. The large peak (at 1580 cm^−1^) close to the N-H stretching peak, is associated with PES. The N-H stretching peak (1545 cm^−1^) was only present in the supported POSS+TMC AL. The broad peak of this AL from 3150–3650 cm^−1^ is likely associated to water and/or unreacted amine groups.

The reason for the suppression of the PA-signal in the supported POSS/polymersomes+TMC AL is not clear. It may be related to the TMC reactivity as discussed before. We used 2 g/L TMC for the supported POSS+TMC AL and POSS/polymersomes+TMC AL, because there was no AL formation at 0.5 g/L. Another TMC concentration may be more optimal for the supported POSS/polymersomes+TMC AL. The potential blockage of PA formation induced by polymersomes should have suppressed the PA formation in the non-supported AL as well, which it did not. However, AL formation was significantly decreased with supported POSS+TMC AL when changing from 2 g/L to 0.5 g/L. Another hypothesis could be that POSS+TMC do not form easily on MF PES. To our knowledge, no former POSS+TMC AL formation on MF PES has been reported. MF PES has significantly bigger pore sizes than PAN. This could hamper the formation of a smooth layer.

**Figure 14:**
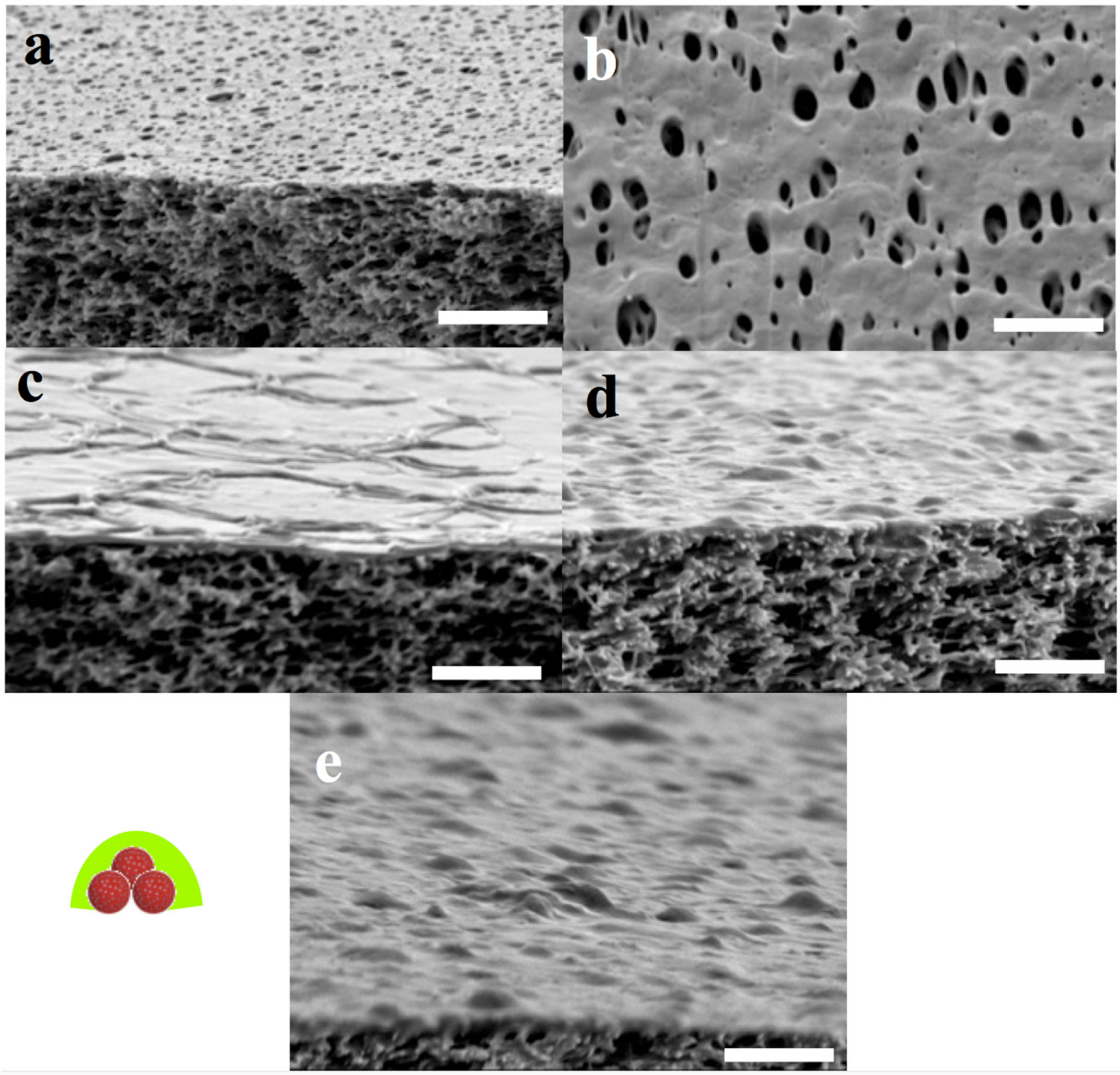
SEM images of MF PES (**a**,**b**) supported POSS+TMC AL on MF PES (**c**) and supported POSS/polymersomes+TMC on MF PES (**d**,**e**). Schematic sketch of polymersome coverage left to (**e**). Micropores of the MF PES were covered completely by the POSS+TMC AL. After addition of polymersomes, small bumps with dimensions similar to the polymersomes were observed on the organic faced side of the AL. Greater bumps may be attributed to accumulations of covered polymersomes. Scale bar is 3 µm.

In contrast to the FTIR analysis, SEM analysis showed a completely covered POSS/polymersomes+TMC AL on the MF PES (Figure 14). In addition, POSS and TMC alone seemed to cover the microporous PES structure completely with the smooth layer, although less defined than for PAN substrates [153]. This could be due to the different pore size as mentioned before. When polymersomes were added, the AL exhibited sub-micron sized bumps. They are 1.5–2 µm in length and 0.5–1 µm in height. Considering a covering AL of 100 nm thickness [153] (Figure 14 sketch in bottom left corner) there would be groups of 6–9 polymersomes in a row in 1–3 layers. In contrast to the non-supported ALs, we can only observe the side facing the organic phase. In the case of the supported POSS/polymersomes+TMC AL, the polymersomes influence the shape of the AL side facing the organic phase to a far higher extent than in the non-supported form. This is most probably due to the different preparations, with regard to POSS/polymersomes being in solution at the non-supported AL formation and being at the water-air-interface or even dried on the MF PES at the supported AL formation. Thus, the chances of polymersomes being integrated in the AL is higher for the supported AL than for the non-supported one.

In conclusion, SEM analysis revealed a successful embedment of polymersomes in a supported AL, whereas FTIR data were less informative. A limitation of FTIR and SEM analysis of supported AL is that only a small fraction of the whole membrane is observed. Another aspect is that the AL could become brittle during drying, and delaminate, or break off when exposed to liquid nitrogen that is used for SEM sample preparation.

We also attempted to test the POSS/polymersomes+TMC AL on MF PES in terms of flux and rejection measurements in FO mode. However, we did not see any FO performance. Approximately one third of the membranes tested were impermeable to salt as evidenced by the low conductivity change in the feed solution within 2 h. The rest of the membranes were leaky as evidenced by an immediate increase in conductivity. The fraction of sealed and leaky membranes of POSS+TMC and POSS/polymersomes+TMC were comparable. In the sealed membranes, the pores are likely clogged by several accumulated layers of POSS+TMC AL. However, after staining with methyl blue no pinholes or scratches were detected on the surface, suggesting that the supporting PES was covered with the AL. As mentioned before, MF PES may not be a suited support for POSS+TMC ALs in general. PAN support did not show any flux without hydraulic pressure, due to the small pore size (5–30 nm) [157]. It may be suited for POSS+TMC for a NF membrane, but not for FO. A compromise would be to use UF PES membranes as used by Lee *et al.* [158].

To conclude this subchapter, we obtained insights in non-supported and supported AL containing POSS with polymersomes in the aqueous phase and TMC in the organic phase. The non-supported POSS/polymersomes+TMC AL was formed successfully with high amounts of polymersomes covered and some of them even integrated inside the AL. The supported POSS/polymersomes+TMC showed a different characteristics. FTIR data indicated a high suppression of the AL formation at polymersome addition, whereas SEM images showed a completely covered and significantly different AL upon polymersome addition. None of the membranes produced, containing POSS and TMC had any reasonable performance, probably due to incomplete coverage of the AL. Still, it was interesting to get an insight into how POSS, TMC and proteopolymersomes are interacting. Further challenges will be to create a functional water separation membrane from these components.

## 5. Perspectives

As mentioned in other reviews [4], ABPMs are rapidly evolving and coming of age. Future challenges will be the upscaling production of both AQPs and block copolymers. Another relevant economic issue is the use of AQP-solubilizing detergents that have a broad price range. ABPMs need to be comparable to established membrane technologies in terms of cost and scale. As seen in Figure 8, all published studies about ABPMs are tested in small-scale laboratory experiments. Even though there are commercially available ABPMs in m^2^-scale, more development will be needed. The lessons learnt from nature are not completely transferred yet.

## Supplementary Files

Supplementary File 1

## Abbreviations/Nomenclature

ABLM: Aquaporin-based lipidic biomimetic membrane
ABM: Aquaporin-based biomimetic membrane
ABPM: Aquaporin-based polymeric biomimetic membrane
7-ADCA: 7-aminodesacetoxycephalosporanic acid
AFM: Atomic force microscopy
AL: Active layer
AQP: Aquaporin
AqpZ: Aquaporin Z
BR: Bacteriorhodopsin
BSA: Bovine serum albumin
CA: Cellulose acetate
CcO: Cytochrome c oxidase
CNT: Carbon nanotube
Cr: Planar shape with protein crystals
DLS: Dynamic light scattering
DMA: Dimethacrylate
DOPE: 1,2-dioleoyl-sn-glycero-3-phosphoethanolamine
DPhPC: 1,2-diphytanoyl-sn-glycero-3-phosphocholine
DTU: Danish Technical University
EFTE: Ethylene tetrafluoroethylene
ELF: Enzyme-labelled fluorescence
EPR: Electron paramagnetic resonance
*f*: Hydrophilic volume ratio
FCS: Fluorescence correlation spectroscopy
FE-SEM: Field-emission scanning electron microscopy
FF-TEM: Freeze-fracture transmission electron microscopy
FhuA: Ferric hydroxamate uptake protein
FI: Functional incorporation
FO: Forward osmosis
FTIR: Fourier-transformed infrared spectroscopy
FR: Film rehydration
*J_s_*: Membrane reverse salt flux
*J_v_*: Membrane water flux
LA: Lipoic acid
LamB: Phage lambda receptor
LbL: Layer-by-layer
*M_n_*: Number-averaged molecular weight
*M_w_*: Weight-averaged molecular weight
MAq: Mixing in aqueous phase
Mc: Micellar shape
MF: Microfiltration
MloK1: Potassium channel from Mesorhizobium loti
MPD: *m*-phenyl diamine
MPEG: Methyl polyethyleneglycol
mPAR: Molecular amphiphile-to-protein-ratio
MOr: Mixing in organic phase
PVL: Polyvalerolactone
NA: Not announced
NAD: *β*-nicotinamide adenine dinucleotide
NADH: Hydrogenated *β*-nicotinamide adenine dinucleotide
ND: Not determined
NF: Nanofiltration
NtAQP1: Tobacco plasma membrane intrinsic protein 1
NtPIP2;1: Tobacco plasma membrane intrinsic protein 2
NUS: National University of Singapore
OG: *n*-Octyl-*β*-D-Glucopyranoside
OmpF: Outer membrane protein F
OmpG: Outer membrane protein G
P: Planar shape
P2VP: Poly-2-vinyl pyridine
P4MVP: Poly-4-vinyl methylpyridine iodide
P4VP: Poly-4-vinyl pyridine iodide
PA: Polyamide
PAA: Polyacrylic acid
PAI: Polyamide imide
PAH: Polyallylamine hydrochloride
PAN: Polyacrylonitrile
PB: Polybutadiene
PBR: Polymer bulk rehydration
PBMA: Polybutyl methacrylate
PCTE: Polycarbonate track-etched
PDA: Polydopamine
PDA: Polydopamine
*P DI*: Polydispersity index
PDMS: Polydimethylsiloxane
PEE: Polyethylethylene
PEOXA: Polyethylene oxazoline
PEO: Polyethylene oxide
PES: Polyethersulfone
PFR: Polymer film rehydration
PGM: Polyglycerol monomethacrylate
PGME: Phenylglycine methyl ester
PHEMA: Polyhydroxyethyl methacrylate
PI: Polyisoprene
PIB: Polyisobutylene
PMOXA: Polymethyloxazoline
POSS: Polyhedral oligomeric silsesquioxane
PPFR: Protein/polymer film rehydration
PPO: Polypropylene oxide
PR: Proteorhodopsin
PRO: Pressure retarded osmosis
PS: Polystyrene
PSf: Polysulfone
PSS: Sulfonate polysulfone
QCM-D: Quartz crystal microbalance with dissipation
RC: Reaction centre
RO: Reverse osmosis
S: Shape
SFLS: Stopped-flow light scattering
SAXS: Small-angle X-ray scattering
SDU: Southern Danish University
SE: Solvent evaporation
SEC: Size exclusion chromatography
SEM: Scanning electron microscopy
SI: Solvent injection
SMTC: Singapore Membrane technology Centre
SoPIP2;1: Spinach plasma membrane intrinsic protein 2;1
TFC: Thin film composite
TMB: 3,3,5,5-tetramethyl-benzidine
TMC: Trimesoyl chloride
UF: Ultrafiltration
V: Vesicular shape
