# Supplementary material for: Aquaporin-Based Biomimetic Polymeric Membranes: Approaches and Challenges
Source: Membranes (Basel). 2015 Jul 31;5(3):307–51. doi: 10.3390/membranes5030307 (PMC4584284; doi:10.3390/membranes5030307)
Supplement: Supplementary File 1 [file membranes-05-00307-s001.pdf]

Aquaporin-Based Biomimetic Polymeric Membranes: Approaches and Challenges

Material and Methods

Materials

Lipids and OG (Anagrade) were purchased from Avanti Lipids, Alabaster, USA. POSS (OctaAmmonium POSS) was purchased from Hybridplastics, Hattiesburg, USA. AqpZ (10 mg/mL in 20 mM Tris pH 8.0, 300 mM NaCl, 300 mM Imidazole and 30 mM OG) and MF PES was kindly provided from Aquaporin A/S. His-tagged AQP10-GFP (50 mM Phosphate, 500 mM NaCl, 250 mM imidazole and 0.75 mg/mL CYMAL-5 at pH 7.5) was kindly provided by Per Amstrup Pedersen lab. All other chemicals except PB-PEO and AQP10-GFP were purchased from Sigma-Aldrich, Brøndby, Denmark and used as received.

PB-PEO Synthesis

PB-PEO was synthesized as described before [2]. Briefly, all used polymers except PB46-PEO32 were synthesized via a one-step anionic polymerization in THF as solvent according to Förster [3]. Bd was polymerized with n-dibutylmagnesium as initiator, where EO was polymerized using 1-*tert*-Butyl-4,4,4-tris(dimethylamino)-2,2-bis[tris(dimethyl-amino)-phosphor-anylidenamino]-2λ^5,4λ^5-catenadi(phosphazene). The polymer was precipitated in cold acetone and analyzed via SEC (for PDI) and NMR (for stochiometry). PB46-PEO32 was synthesized via two-steps after Hillmyer [4]. After Bd polymerization in the same way, the polymer was precipitated and brought back in solution for EO polymerization with potassium naphthalenide as initiator. Precipitation and analysis was done the same way. For stock solution, polymer was dissolved in chloroform at a concentration of 10mg/mL and stored at −20 °C until use.

Proteo- and Polymersomes Preparation via FR

Polymerstock was injected in a round flask and evaporated at room temperature at approximately 125 rpm for several hours. For polymersome preparation, the film was rehydrated with Tris buffer (10 mM Tris pH 8.0, 50 mM NaCl) with 13 mg/mL OG, for proteopolymersomes the film was rehydrated with AqpZ stock and Tris buffer with 13 mg/mL OG and left stirring for 12 h at room temperature. Then, four times the rehydration volume of Tris buffer was added to end up at a polymer concentration of 25 mg/mL. At the same time, 20 mg per mL solution was added and the sample was left shaking for 3 h at room temperature and another 12 h at room temperature after addition of another portion of the same amount of biobeads. The final polymer concentration was 25mg/mL.

FF-TEM

FF was done with a MED020 with EM VCT100 shuttle attached (Leica, Wetzlar, Germany). A 3 mm aluminium sample carrier was filled with 1.2 µL of 25 mg/mL proteo- or polymersomes in its deeper pit with 300 µm depth. Another sample carrier was placed on top with the 200 µm towards the sample drop. The sandwich was shock-frozen by plunging it in liquid ethane. It was fixed in a sample holder under liquid nitrogen atmosphere and brought in a vacuum chamber at −140 °C. There, the lower carrier with

the 200 µm pit towards the sample was removed abruptly to enable a clean crack through the sample. The sample was coated with 2 nm carbon and 4 nm platinum at 45° tilting and 19 nm backing layer without tilt. Outside the chamber, the replica was thawed for 5 min and placed carefully in a bath of 100 mg/mL OG to solubilize the thawed vesicles. The replica, swimming on the surface, was caught on a uncoated 400 Mesh copper TEM grid (Agar scientific, Essex, UK).

TEM was done on a CM100 (Philips, Amsterdam, The Netherlands) with an Veleta 2k CCD camera (Olympus, Shinjuku, Japan). The applied voltage on a tungsten source was 80 kV with a 100 µm objective sense aperture.

SAXS

Prior to SAXS measurements, 20 mg/mL proteo- or polymersomes were extruded 20 times through 200 nm pore sized tracketched polycarbonate membranes and centrifuged at 20,000 rpm for 10 min to remove aggregates. Measurements were carried out at the beamline I911-4 at MAXLAB synchrotron in Lund, Sweden. The 2D detector images were radially averaged and background subtracted using the dedicated software at the beamline. Absolute intensity callibration to units of cm^{-1} was done using water as a scattering standard.

FCS

25 mg/mL of proteopolymersomes were extruded 20 times through 200 nm pore sized track-etched polycarbonate membranes and just before measurement centrifuged at 10,000 rpm for 10 min to remove any aggregates that could disturb the sensitive FCS measurements. FCS was performed with an FLIM confocal microscope (PicoQuant, Berlin, Germany) with an excitation length of 482 nm and a laser pulse frequency of 400 MHz during 600 s. Proteopolymersomes were diluted to 10 nM GFP concentration. 20 µL of samplewere set on a cleaned specimen glass. For optimal measurement, the confocal volume was focussed 20 µm inside the sample volume.

DLS

DLS was performed on a Nano Zetasizer (Malvern, Worcestershire, UK). 1 mL of 25 mg/mL polymersomes were introduced in a cuvette and measured 3 × 6 times for 10 s.

Non-supported AL Formation

The polymersome sample was sonicated for 5 min. Approximately 20 mL of aqueous phase (9 g/L POSS adjusted with 1M NaOH to pH 9.5 in MilliQ with 0.4 g/L polymersomes if they were present) was poured in a beaker. Afterwards 20 mL of organic phase (0.5 g/L TMC in n-hexane) was added carefully. After 5 min, the AL was collected with a small spoon or a spatel and put on a petri dish to air dry and later in the vacuum oven to dry at 50 °C for at least 12 h.

Formation of Supported AL Containing POSS and TMC

Supported ALs containing POSS and TMC were processed following Dalwani *et al.* [5]. A perforated metal frame with an effective area of 13.86 cm^2 was taped with double sided tape. A cellulose-based

highly porous support was placed on top. Another layer of tape was put and the MF PES was set on top with the dense side up. This sandwich was connected to a vacuum pump and placed in a bath with 0.5 g/L SDS in MilliQ to wet the membrane. This solution was then soaked throught the MF PES at 0.5 bar pressure for 10min. The frame was then placed in another batch containing the aqueous phase (9 g/L POSS adjusted to pH 9.5 using 1 M NaOH in MilliQ, if they are present 0.4 g/L polymersomes), whereafter it was again soaked for 10 min at 0.5 bar. It was then put in the organic phase (2 g/L TMC in n-hexane) and allowed to react for 5 min (no vacuum soaking). The frame was removed from the bath and leftover TMC was rinsed with n-hexane. For J_v and J_s testing, the membrane was placed in a parafilm-taped petri dish with MilliQ until use. For FTIR and SEM characterization, a piece was cut out and vacuum dried at 50 °C for 12 h.

FTIR

FTIR was performed on a Tensor 27 (Bruker, Billerica, USA) on a ZnSe crystal at room temperature. For each sample, an average of 32 scans per measurement were taken. Further analysis was done using Excel.

SEM

SEM analysis was performed on a JSM-6010LA (JEOL, Tokyo, Japan) with 5 kV electrons from tungsten source and a working distance of 10 mm.

Microfluidic Approach

The microfluidic approach was performed like described in Zhang et al. [6]. The chip was produced on a silicon wafer by photolithography and ion etching and bonded to a glass wafer. The inner walls of the compartments were chemically hydrophobized using silicon oil. The chip was placed in a self-made holder with reservoir connections. The aqueous phase (9 g/L POSS pH 9.5, 0.4 g/L polymersomes in MilliQ) was introduced within applying a pressure of 2400 Pa. The organic phase (0.5 g/L TMC in hexane) was then introduced with a minimal introducing pressure. To allow AL formation without pressure differences, all connections were opened after introduction of both phases. The AL formation was monitored with a AxioCam MRc 5 camera that was connected to a Axiovert 40 optical microscope (Carl-Zeiss, Jena, Germany).

J_s and J_v Testing of Supported AL

The supported AL, as well as the AIM was tested for J_v and J_s using an own-built measurement system for two parallel measurements. A small chamber (3.92 cm^2 for supported AL containing POSS and TMC) was connected with hard plastic tubes to the draw solution (1 L 1 M NaCl in MilliQ) on a balance and the feed solution (1 L MilliQ) as well as to the pumps. The speed velocity was set to 10 mL/min in cross-flow mode. The membrane in the chamber was placed with AL up (FO mode). Weight increase and conductivity was measured manually approximately every 15 min for at least 2 h or longer if there was a reasonable weight increase of the draw solution. Further analysis was done with Excel.

References

1. Bomholt, J.; Hélix-Nielsen, C.; Scharff-Poulsen, P.; Pedersen, P.A. Recombinant Production of Human Aquaporin-1 to an Exceptional High Membrane Density in Saccharomyces cerevisiae. *PLoS One* 2013, doi:10.1371/journal.pone.0056431.
2. Habel, J.; Ogbonna, A.; Skaarup, N.; Schulte, L.; Almdal, K.; Hélix-Nielsen, C. How molecular internal-geometric parameters affect PB-PEO polymersome size in aqueous solution. *Phys. Chem. Chem. Phys.* **2015**, To be submitted.
3. Förster,S.; Krämer, E. Synthesis of PB-PEO and PI-PEO Block Copolymers with Alkyllithium Initiators and the Phosphazene Base *t*-BuP_4. Macromolecules, **1999**, *32*, 2783–2785.
4. Hillmyer, M.A.; Bates, F.S. Synthesis and Characterization of Model Polyalkane-Poly(ethylene oxide) Block Copolymers. *Macromolecules* **1996**, *29*, 6994–7002.
5. Dalwani, M.; Zheng, J.; Hempenius, M.; Raaijmakers, M.J.T.; Doherty, C.M.; Hill, A.J.; Wesslinge, M.; Benes, N.E. Ultra-thin hybrid polyhedral silsesquioxane–polyamide films with potentially unlimited 2D dimensions. *J. Mater. Chem.* **2012**, *22*, 14835–14838.
6. Zhang, S.; Peh, M.H.; Thong, Z.; Chung, T.S. Thin Film Interfacial Cross-Linking Approach To Fabricate a Chitosan Rejecting Layer over Poly(ether sulfone) Support for Heavy Metal Removal. *Ind. Eng. Chem. Res.* **2015**, *54*, 472–479.

© 2015 by the authors; licensee MDPI, Basel, Switzerland. This article is an open access article distributed under the terms and conditions of the Creative Commons Attribution license (http://creativecommons.org/licenses/by/4.0/).
